# Pronounced interplay between intrinsic phase-coexistence and octahedral tilt magnitude in hole-doped lanthanum cuprates

**DOI:** 10.1038/s41598-022-18574-1

**Published:** 2022-08-22

**Authors:** Jeremiah P. Tidey, En-Pei Liu, Yen-Chung Lai, Yu-Chun Chuang, Wei-Tin Chen, Lauren J. Cane, Chris Lester, Alexander N. D. Petsch, Anna Herlihy, Arkadiy Simonov, Stephen M. Hayden, Mark Senn

**Affiliations:** 1 Department of Chemistry, University of Warwick, Gibbet Hill, Coventry, CV4 7AL UK; 2 Department of Physics, Tamkang University, Tamsui, 25137 Taiwan; 3 Center for Condensed Matter Sciences and Center of Atomic Initiative for New Materials, National Taiwan University, Taipei, 10617 Taiwan; 4 National Synchrotron Radiation Research Center, Hsinchu, 30076 Taiwan; 5 Taiwan Consortium of Emergent Crystalline Materials, Ministry of Science and Technology, Taipei, 10622 Taiwan; 6 H.H. Wills Physics Laboratory, University of Bristol, Bristol, BS8 1TL UK; 7 ISIS Neutron and Muon Facility, Rutherford Appleton Laboratory, Didcot, OX11 0QX UK; 8 Department of Materials (Multifunctional Ferroic Materials), ETH Zürich, Vladimir-Prelog-Weg-5/10, 8093 Zurich, Switzerland

**Keywords:** Superconducting properties and materials, Phase transitions and critical phenomena, Structure of solids and liquids, Superconductors, Solid-state chemistry

## Abstract

Definitive understanding of superconductivity and its interplay with structural symmetry in the hole-doped lanthanum cuprates remains elusive. The suppression of superconductivity around 1/8th doping maintains particular focus, often attributed to charge-density waves (CDWs) ordering in the low-temperature tetragonal (LTT) phase. Central to many investigations into this interplay is the thesis that La_1.875_Ba_0.125_CuO_4_ and particularly La_1.675_Eu_0.2_Sr_0.125_CuO_4_ present model systems of purely LTT structure at low temperature. However, combining single-crystal and high-resolution powder X-ray diffraction, we find these to exhibit significant, intrinsic coexistence of LTT and low-temperature orthorhombic domains, typically associated with superconductivity, even at 10 K. Our two-phase models reveal substantially greater tilting of CuO_6_ octahedra in the LTT phase, markedly buckling the CuO_2_ planes. This would couple significantly to band narrowing, potentially indicating a picture of electronically driven phase segregation, reminiscent of optimally doped manganites. These results call for reassessment of many experiments seeking to elucidate structural and electronic interplay at 1/8 doping.

## Introduction

The prospect of room-temperature superconductivity (SC) has provided a tantalising target^1^ to chemists and physicists alike for some 35 years since the discovery of high-temperature SC in La_2−*x*_Ba_*x*_CuO_4_. Yet, despite the extensive research efforts drawn by this and the range of compounds that followed, understanding of the origin and mechanism of the SC and competing electronic phenomena, including charge-density wave (CDW) and spin-ordered (SO) states, remains limited to the electronic context^2,3^ and unification with the structural behaviour has proved elusive^4^. In this context, a comprehensive understanding of the microscopic structure–property relationship in these compounds is highly desirable and would further illuminate the path to enhanced critical temperatures (*T*_C_) for three-dimensional (3D) SC in these and similar materials.

**Figure 1:**
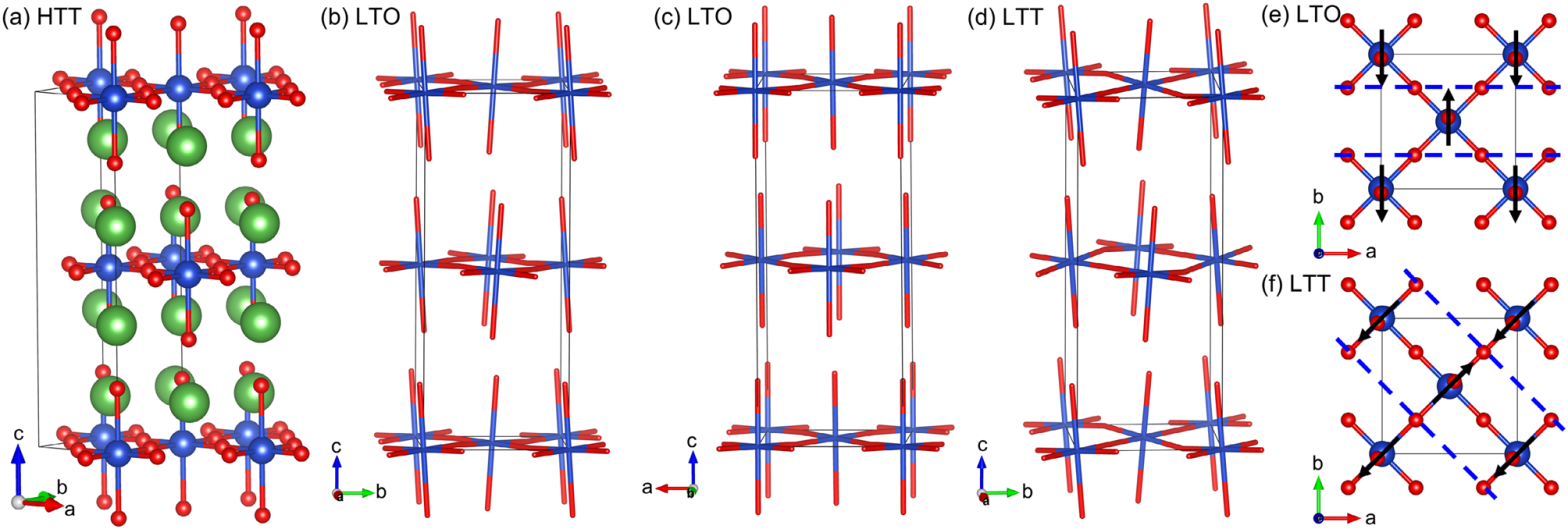
(**a**) Representation of the HTT structure of the Ruddlesden-Popper *n* = 1 cuprates, packed according to the low-temperature supercell and showing its relationship to the *I*4/*mmm* aristotype unit-cell (for LTO and LTT, **a** = **a**_**HTT**_ − **b**_**HTT**_**, b** = **a**_**HTT**_ + **b**_**HTT**_ and** c** = **c**_**HTT**_); *A*-site cation (*e.g.*, La, Ba,…) in green, copper (*B*-site) in blue and oxygen in red. (**b**) and (**c**) show representations of the LTO structure (*A*-site cations omitted for clarity) viewed just off (**a**) and (**b**), respectively. (**d**) Presents a similar depiction of the LTT phase viewed just off (**a**). Note how, in LTO (**b**, **c**), the sense of the distortion is such that they propagate solely along (**b**) and all Cu–O–Cu bonds are buckled while, in the LTT phase (**d**), distortions propagate along alternating diagonals in adjacent layers, [1 1 0] at *z* = 0 and [1 −1 0] at *z* = 0.5 with respect to the super-cell, and so buckle only half the Cu–O–Cu bonds, along orthogonally alternating vectors in each layer. (**e**) and (**f**) show the CuO_6_ octahedra in the *ab* planes of the LTO and LTT phase, respectively, viewed parallel to (**c**); black arrows depict the direction in which the tilts occur, *i.e.*, their ‘sense’, and the blue dashed lines the associated ‘buckles’ in the CuO_2_ planes.

At high temperature, the Ruddlesden-Popper *A*_*n*+1_*B*_*n*_O_3*n*+1_ (*n* = 1) cuprate perovskites crystallise in the high-temperature tetragonal (HTT), *I*4/*mmm* phase (Fig. 1a), wherein only two internal degrees of freedom are allowed, perturbing the *A*-site cation and apical oxygen atoms along the crystallographic *c* axis^5^. In general, as these samples are cooled, they undergo a phase transition (defined as occurring at *T*_LTO_) to an orthorhombic, *Bmab* supercell (basis w.r.t HTT: ([1 −1 0], [1 1 0], [0 0 1]) with no change of origin). In this so-called low-temperature orthorhombic (LTO) phase (Fig. 1b,c), additional degrees of freedom are realised which transform as the irreducible representation (irrep) X_3_^+^(a;0) of *I*4/*mmm* and correspond to the *B*O_6_ octahedra tilting off the crystallographic *c* axis by rotation about the *a* axis of the low-temperature supercell (Fig. 1e). This results in all Cu–O–Cu bonds buckling to an equal degree in an alternating fashion above and below the (2 0 0) planes. It is in this LTO phase that 3D SC is understood to arise at certain doping levels of the parent lanthanum cuprate phase.

In the case of La_2−*x*_Ba_*x*_CuO_4_, the temperature/doping phase diagram exhibits two pronounced regions of superconductivity, with *T*_C_ peaking at *c.a. x* = 0.095 and 0.155, either side of a pronounced dip at 1/8th doping^6^. This suppression of *T*_C_ is understood to be coincident with the occurrence of a second structural phase transition, with *T*_LTT_ commencing below 80 K and forming the low-temperature tetragonal (LTT) phase (*P*4_2_/*ncm*) that may be expressed in the same supercell setting as for LTO. In this arrangement, tilt degrees of freedom now correspond to an order parameter direction of X_3_^+^(a;a), wherein the sense of the octahedral tilting is described by equal magnitudes in both **a** and **b** with respect to the low-temperature supercell (Fig. 1f) and the resulting rotations propagating along orthogonally alternating diagonals in adjacent CuO_2_ layers (Fig. 1d). This results in only half of the Cu–O–Cu interactions—those along the [1 1 0] and [1 −1 0] directions of the supercell for the different layers centred at *z* = 0 and ½—buckling out of the (2 0 0) planes with O atoms alternating above and below the CuO_2_ plane. Perpendicular to these within each layer, the Cu–O–Cu interactions adopt linear configurations that form 1D chains. It is important to note that, although 3D SC is typically suppressed in the LTT phase, two-dimensional (2D) superconductivity is understood as being able to coexist with the CDW state^7^.

Unlike the second-order HTT → LTO phase transition, which in La_2−*x*_Ba_*x*_CuO_4_ (*x* = 0.125; LBCO) occurs upon cooling below ~ 210 K^8^, there is no group-subgroup relationship between the LTO and LTT phases, requiring the phase transition to be first-order in nature. A second-order transition pathway could only be envisaged if the system passed continuously through a lower symmetry phase that formed a subgroup of both LTT and LTO phases. In this context, evidence of such an intervening ‘low-temperature, less-orthorhombic’ (LTLO) phase (in *Pccn*) has been seen in rare-earth doped systems, including La_2−*y*−*x*_Nd_*y*_Sr_*x*_CuO_4_^9^ and La_2−*y*_Sm_*y*_CuO_4_^10^. Surprisingly, these transitions still appear to proceed with clear elements of first-order character and pronounced coexistence of orthorhombic phases, presenting further unexplained complexities to the structural behaviour of this family of materials.

Coincident with the occurrence of the LTT phase in LBCO is the formation of a modulated charge density wave (CDW) state (*i.e.*, *T*_LTT_ = *T*_CDW_ = ~ 55 K)^6^, although these are not necessarily mutually inclusive in these systems. In the case of La_2−*x*−*y*_Eu_*y*_Sr_*x*_CuO_4_ at *x* = 0.125 and *y* = 0.2 (LESCO), although the structural phenomena observed are analogous to those of LBCO, *T*_LTT_ resides at higher temperature (~ 130 K) and the structural transition no longer coincides with *T*_CDW_. Here, the charge-ordering transition is widely reported as being *ca*. 80 K^11^, although reports have emerged of there being some degree of order present at temperatures greater even than *T*_LTT_^12,13^.

Theories that have arisen surrounding the suppression of SC at around the 1/8th doping level have largely centred around the observation of charge-stripe pinning, *i.e.*, the static localisation of CDWs, in the LTT phase^14^. It has been postulated that these may become immobilised by domain walls in the LTT phase and, in the context of multi-phase systems, may then further extend into LTO domains^15^. Taking this further is that understanding that the spatial arrangement of these stripes, as affected by the LTT symmetry, results in the vanishing of interlayer Josephson coupling^16^ which can otherwise explain the transformation of 2D to 3D superconductivity in these systems^17^.

In the context of the structural phase transitions, two key positions have seen attention. The first of these is that they are purely structural in origin, *i.e.*, they are the response to solely a mismatch between *B–*O and *A*–O bond lengths and to the variance of the latter. In this context, the observation of the LTO/LTT structural transition in electronically simplified systems provides evidence that electronic considerations are not completely necessary to the transition. It logically follows this position that electronic ordering is then susceptible and responsive to structure^18,19^ but, more than this, it is so in a non-commutative fashion. In apparent opposition to this, it has also been proposed that the LTO/LTT phase transition is necessarily electronically driven in the lanthanum cuprates^20^. In our recent study, we show by use of a plain band insulator surrogate, La_2_MgO_4_, that the transition can indeed be driven solely by structural means, but argue from DFT results that the energies involved are sufficiently small as to be susceptible to contributions from electronic ordering which may then act to select the resulting form^21^. This idea, that the resulting phases arise from a complex interplay of structural and electronic degrees of freedom, has itself also seen distinct discussion^22^.

Thus, despite extensive experimental and theoretical interrogation, the specific mechanisms of interplay and competition between structural symmetry, CDWs and SC remains largely speculative. This is in part due to an incomplete understanding of the origin of the CDW state and associated structural distortions. However, as we will show, this has likely also been hampered by an incomplete understanding of the phase (and symmetry) in which the CDW state arises.

The greatly enhanced *T*_CDW_ of LESCO and its apparent separation from *T*_LTT_ presents it as an attractive prototype for the study of structure–property relationships in this class of compound. In extension to this, while there was discussion earlier in the development of this field surrounding a persistence of the LTO phase into the LTT regime for other cuprates, including LBCO^8,23^ and indeed lower degrees of Eu doping than investigated herein^24^, it has widely been accepted that this ceases to be the case^19^ at a Eu content of *y* = 0.2, *i.e.*, that LESCO adopts a wholly LTT state at low temperatures. This property in particular has seen the system become prominent in the study and interpretation of competing electronic phenomena in the hole-doped lanthanum cuprates and, thus, it forms the focus of the present report.

Notably, attention has been given recently to the photo-induction of long-lasting SC in LESCO^25^. At 10 K, a signal associated to 3D superconductivity was observed within ~ 1/10th the time period of the thermally available phonon modes that would otherwise allow relaxation of the LTT tilt system and a recovery of symmetries typically deemed compatible with the phenomenon^26^. The rapid onset of the signal thus appeared incompatible with the present understanding of the symmetry of the LTT phase, its CDW state and that required for SC. Subsequent investigations into the relaxation^27^ of the photoinduced state and replicating the result in LBCO^28^ continued to presume fully LTT macroscopic structures while tendering developments in the proposed interplay of the LTT structure, CDW and superconducting states. However, it bears mentioning that there has also been discussion of intrinsic phase inhomogeneity in predominantly LTT phases of La_1.83−*x*_Eu_0.17_Sr_*x*_CuO_4_ potentially being the origin of superconductivity at doping up to around *x* = 0.15^24^.

We report herein a detailed, combined single-crystal and high-resolution powder X-ray diffraction study of LESCO, LBCO and LSCO (La_2−*x*_Sr_*x*_CuO_4_ at *x* = 0.125). Detailed analysis of the structural composition is presented and serves to show that the samples of LESCO and LBCO studied herein each adopt an intrinsic phase-coexistence of both LTT and LTO structures at low temperature, contrary to the prevailing interpretation in the literature of the ground state structures being purely LTT. With this knowledge of the intrinsic phase segregation in those phases, we fully model our high-resolution diffraction data and reveal the magnitude of the octahedral tilting to be substantially underestimated in previous discussions of the non-superconducting LTT phases, themselves widely taken as the origin of the suppression to SC in LESCO and LBCO. In contrast, no such revision of the structural understanding is found for LSCO, and we speculate that this may be linked to the persistence of SC in this system, itself atypical for systems of doping *x* = 0.125. The strong coupling that we observe in these 1/8th-doped cuprates between tilt magnitude and structural symmetry point towards an electronic origin of the intrinsic phase coexistence.

## Results and discussion

### Phase coexistence

**Figure 2:**
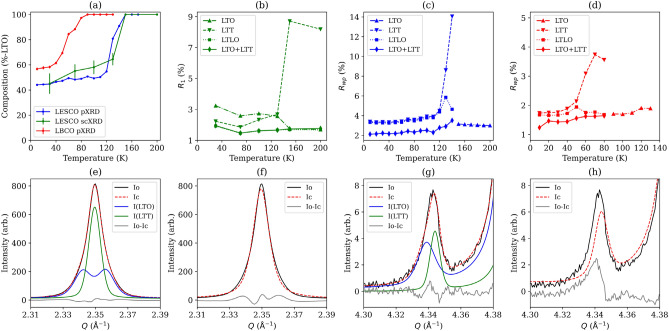
Top left to right, plots of: (**a**) the refined percentage of the LTO phase in the LESCO scXRD and pXRD and in LBCO pXRD data, showing errors as estimated by *SHELX* and *TOPAS* (error bars drawn at 3σ confidence level; a discussion of the accuracy of these is provided in the Methods section); (**b**) *SHELX* outputted *R*_1_ fit-factor for all reflections for the LTT, LTO, LTLO and LTO+LTT single-crystal refinements of LESCO; and the *R*_wp_ fit-factors for the LTT, LTLO, LTO+LTT and LTO models for the pXRD of (**c**) LESCO and (**d**) LBCO. Bottom left to right, plots of pXRD refinement fits of: (**e**) the dual-LTO+LTT and (**f**) single-LTT models to the (2 0 0)/(0 2 0) peak set at 50 K for LESCO; and the same models for (**g**) and (**h**), respectively, fit to the (2 3 2)/(3 2 2) peak set for LESCO. For (**e**) and (**g**) we show explicitly the two components that contribute to the overall calculated trace; equivalent plots for LBCO in Supplemental Fig. 9 and include a reflection of class *hhl* for comparison.

We demonstrate in the methods section that the combination of laboratory single-crystal (Fig. 2b; see also Supplemental Fig. 1–8) and high-resolution synchrotron powder (Fig. 2c–g; see also Supplemental Fig. 9) X-ray diffraction (scXRD and pXRD, respectively) unambiguously points towards a pronounced phase coexistence in LESCO between the LTO and LTT phases, designated the LTO+LTT model. This result stands contrary to conventional wisdom where LESCO has been used as a prototype system for the study of the CDW-state based on the belief that it adopts a purely LTT-state at low-temperature. The phase coexistence evolves from 45(3)% at 30 K to 64.5(16)% at 130 K in the single crystal, corroborated by Rietveld refinement results against the powder diffraction data giving 44.1(2)% at 10 K, through to 54.73(15)% at 120 K and again achieving a solely LTO state in both by 150 K (see Fig. 2a). The phase coexistence is considered as being intrinsic, further verified by single crystal diffraction on a number of crystallites for which approximately equal phase fractions were found (Supplemental Table 1). Furthermore, the bulk by pXRD clearly evidences a single, highly crystalline phase above *T*_LTT_ (*e*_0_ = 0.034% and 0.037% at 200 K for LESCO and 130 K for LBCO, respectively; see Supplemental Fig. 10), precluding any chemical inhomogeneity as the origin for this phase segregation.

Studies on other rare-earth co-doped La_2−x−*y*_RE_*y*_Sr_*x*_CuO_4_ have established regions of phase coexistence to be a fairly common feature of the LTO-to-LTT phase transition, particularly in the context of Nd-doping for which there are reasonable claims of an intervening LTLO system^9,29^. What is noteworthy is that, while samples with a Eu content of up to *y* = 0.17 and across a range of *x*-doping are indicated to exhibit phase segregation in the LTT regime^24,30^, this is understood to have ceased^19^ by *y* = 0.2. It is this supposed simplicity of form that has provided such focus to LESCO, *i.e.*, the *x* = 0.125, *y* = 0.2 composition we study herein, in the context of structure–property studies in these systems. Such a claim is in clear opposition with the results we report, which show that low-temperature data for LESCO should be interpreted in the context of a considerable coexistence of the LTO and LTT phases. Remembering that the ground-state of the LTO phase is most commonly superconducting but proposed to accommodate pinned-CDWs where they protrude from adjacent LTT domains^15^, a revisitation of the discussions surrounding bulk electronic phenomena therein, such as the rapid photoinduction and slow relaxation of SC, and the associated implications for structure–property relationships would thus seem warranted.

This observation of such overlooked complexity at the 1/8th doping level, along with indications of such complexities within the literature at other doping levels, would appear to support a view that such structural interplay may be intrinsic to these systems at large, concomitant with the competition between the CDW and SC states. With this in mind, we expand our study to revisit the behaviour in other well-discussed compositions.

The combined scXRD and pXRD study on LESCO allows us to develop a robust Rietveld refinement protocol that we implement against equivalent high-resolution pXRD data for LBCO. Similar to the case of LESCO, we find a protracted phase coexistence between the LTT and LTO phases in LBCO down to the lowest temperatures studied (See Fig. 2a), again contrary to the widely held view of this phase (see Supplemental Fig. 9 for plots of pXRD Rietveld refinement fits to key reflections). In this case, we find 56.7(12)% LTO existence at 10 K, evolving to 69.4(16)% at 50 K prior to the discernible onset of the transition to a solely LTO phase which is complete by ~ 80 K.

The observation of the LTO and LTT phases coexisting in the low-temperature regime of *n* = 1 Ruddelsden-Popper perovskite cuprates is not itself a novel result. Already in 1989, reports of the LTT and LTO phases coexisting in samples of La_2-*x*_Ba_*x*_CuO_4_ across a range of dopant levels were being made, albeit with significant variation in the reported compositions. Cox et al. reported the apparent persistence of the LTO phase below *T*_LTT_ for La_1.9_Ba_0.1_CuO_4_ [~ 10% LTO at 15 K]^31^. Axe et al. found significantly higher LTO composition for the aforementioned material [~ 65%] and reported a phase-fraction for LBCO (*i.e.*, *x* = 0.125) of ~ 55% LTO in their low-temperature X-ray data^8^, agreeing well with the levels we find herein. This result later saw further disagreement from a qualitative assessment of peak shape [< 15% LTO at 15 K]^23^ and similarly in a neutron PDF study [~ 5% LTO at 10 K]^32^*,* although this observation does not seem to have held traction in the continued discussion of the system.

While there are a few experiments that favour an interpretation that phase coexistence is not intrinsic, this may be a consequence of data collection methodology. In the case of LBCO, key support of single-phase behaviour below *T*_LTT_ appears to be given in single-crystal rocking curves^33^. Here, no evidence of a twinned orthorhombic system is reported to have been found at 10 K when interrogating the (200)/(020) reflections of the low-temperature phases. It is tempting to suggest that this may originate from very high beam energies (100 keV) affecting the LTO-to-LTT conversion, particularly if electronic interplay contributed to the structural energy landscape. Indeed, we find in our own experimentation that intense irradiation can push the transition significantly towards completion for single-crystals of LESCO (Supplemental Fig. 2). At lower average *A*-site radius than we span in our present study, *i.e.*, for La_1.6−*x*_Nd_0.4_Sr_*x*_CuO_4_, some disagreement between powder diffraction^9,15^ and rocking curves^34^ also seems to be present in the literature. However, these rocking curve experiments are performed at a neutron source, suggesting that an alternative explanation for this particular discrepancy must exist. Nevertheless, we highlight that our single-crystal study confidently observes the loss of the *hkl*≡*khl* tetragonal equivalence in supercell reflection intensities for LESCO at all temperatures below *T*_LTT_ (Supplemental Figs. 3–8), and three peaks owed the two LTO twin components and the LTT phase are also evident at 88 K in high-resolution single-crystal experiments (Supplemental Fig. 2). It is then compelling to find repetition of the according indications of this behaviour seen in our LESCO powder diffraction study throughout the low-temperature regime of that for LBCO. Our results thus concretely demonstrate the phase coexistence in LESCO while reinstating uncertainty over the low-temperature structural identity of LBCO and potentially other doped lanthanum cuprates. Further work is clearly required to understand what affects this apparent inconsistency in the phase behaviour of these systems, and local probe techniques, such as NMR-NQR^35,36^, may help shed light in this context.

Since neither LBCO nor LESCO exhibit 3D SC at the 1/8th doping level, one might reasonably attribute this to the prevalence of the LTT phase. To verify that the same phase coexistence is not evident in systems with clear onset of SC, we study also La_2−*x*_Sr_*x*_CuO_4_ at doping *x* = 0.125. LSCO is understood to exist solely in the LTO phase at low temperatures and for which only minor suppression of *T*_C_ is observed around the 1/8th doping level^37^. The possibility of the LTT phase existing as a minor component alongside that of LTO has been proposed as a possible cause for this slight dip in *T*_C_^15^ but, to our knowledge, observations to support this have been limited to those of a dynamic nature^38^. Moreover, studies on closely doped systems would indicate that, were there to be a commencement of the LTO → LTT transformation, this would occur well below *T*_C_^39^, indicating the suppression not to be owed to the simple existence of an LTT phase. Here, our scXRD and pXRD show unambiguously that a single phase LTO model describes the data best, as outlined in the methods section. This is established by powder diffraction down to 15 K, well within the region of SC for LSCO, and more rigorously by both powder and single-crystal data at 30 K, a temperature locating in the non-superconducting regime about the aforementioned dip in *T*_C_. Further measurement at 50 K, a temperature significantly above *T*_C_ yet within the recently observed short-range charge-ordered regime^40^, also provides no indication of an LTT phase. Thus, the data further discredits the proposition of LTT-formation as being the origin of the slight suppression of superconductivity in LSCO. We note that, while we cannot fully rule out a subtle monoclinic distortion for this case or those of LESCO and LBCO, as has been reported for other doping compositions^41–43^, such symmetry breaking should represent only a minor perturbation to the limiting LTT and LTO descriptions discussed herein.

### Structural analysis

Next, we precisely investigate the structural implications of this hitherto unaccounted-for phase coexistence on the reported structures for LSECO and in turn LBCO for both the refined LTO and LTT models across the phase-coexistent region. We emphasise that, because previous work has essentially failed to identify the dual phase regime, those models have been fit to an averaged set of intensities derived from the experimental sum of the squared LTO and LTT structure factors. This results in an incomplete picture of the structural geometries at play, particularly for the relative magnitude of buckling in the CuO_2_ planes.

**Figure 3:**
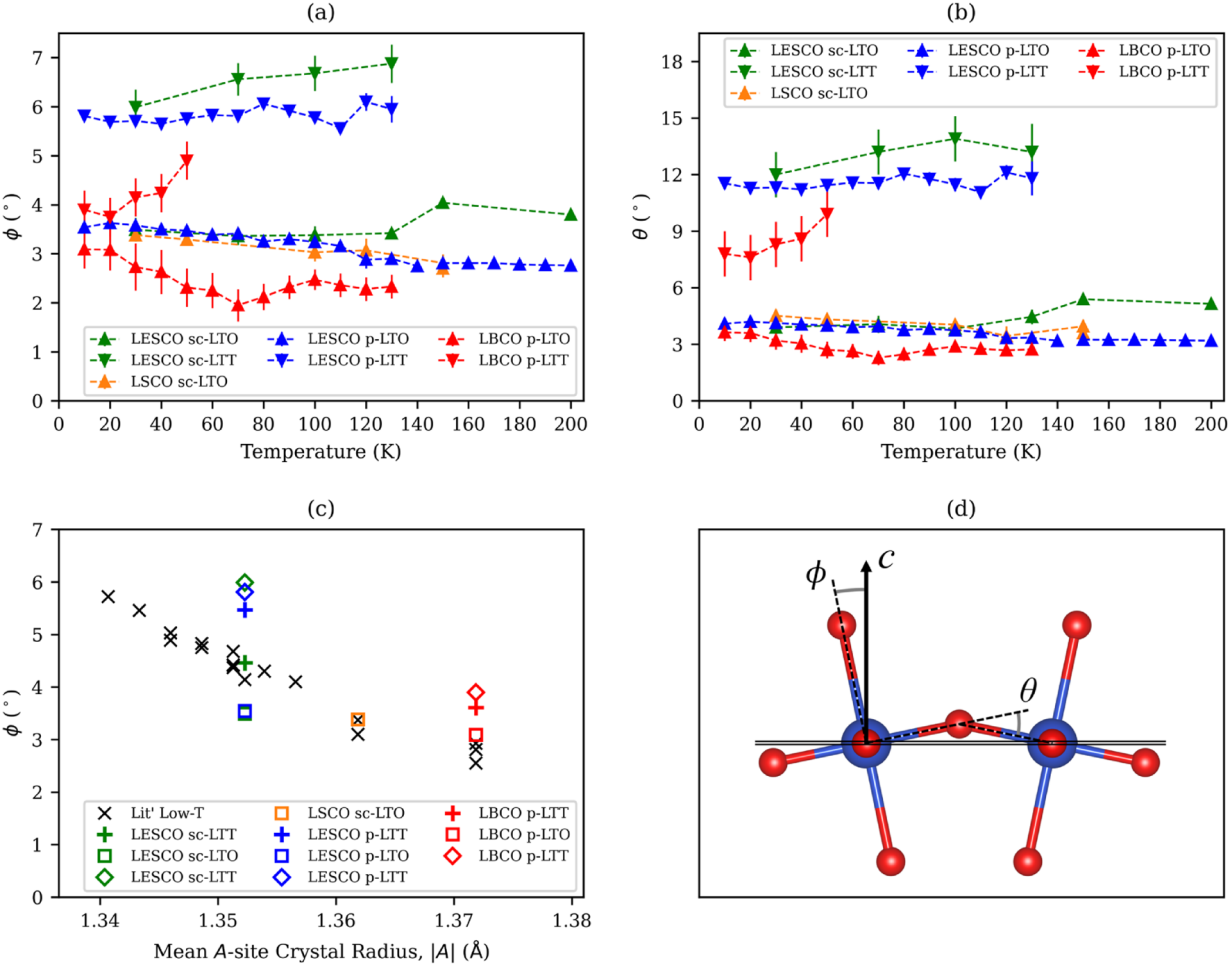
Plots of (**a**) the octahedral tilt angles, *φ*, and (**b**) Cu–O_eq_–Cu’ bond buckle, *θ*, for the appropriate refinements of both pXRD (“p−”) and scXRD (“sc−”)data sets with error bars drawn at 3σ confidence level; (**c**) *φ* as a function of the average crystal radii^44^ of the doped *A*-site found in the literature^10,23,45–47^ for (La,Nd,Eu)_1.875_(Ba,Sr)_0.125_CuO_4_ (*y* = 0.2–0.8 for Nd, 0.2 for Eu), those obtained for the LTO+LTT and single-phase LTT models for LESCO and LBCO, and LTO mode for LSCO, discussed in the text. (**d**) Diagram of two corner-sharing CuO_6_, viewed in the equatorial plane, showing the identity of angles *θ* and *φ*. We omit LTT parameters in (**a**) and (**b**) where the phase fraction falls below 15% and their refinement grows unreliable. See Supplemental Figs. 11 and 12 for symmetry adapted distortion mode amplitudes and cell parameters, respectively.

It has long been assumed that the transformation from the LTO to LTT structure serves negligible impact on the magnitude of the octahedral tilting. However, we find a marked difference between the tilt angle, *φ* (Fig. 3d), of the phases below *T*_LTT_, with *φ*_LTO_ = 3.54(4)° and *φ*_LTT_ = 5.81(4)° in LESCO and being more or less temperature independent below *T*_LTT_ (Fig. 3a). Comparing the geometry of the CuO_2_ planes explicitly, the difference between phases is even more stark and, in the LTO phase, the bridging Cu–O–Cu angles buckle (*θ*; Fig. 3d) by ~ 3–4° out of the plane while, in the LTT phase, half of the in-plane Cu–O–Cu angles are buckled out of the plane by between 11 and 14° (Fig. 3b; the rest remain necessarily linear). A similar case is seen in the analysis of LBCO, albeit to a lesser extent, with the arguably more physically significant CuO_2_ buckling (vide infra) amounting to *θ*_LTO_ = 3.63(15)° and *θ*_LTT_ = 7.8(4)° at 10 K for octahedral tilts of *φ*_LTO_ = 3.09(13)° and *φ*_LTT_ = 3.90(13)°.

The comparison of our results with values of *φ* for 1/8th-doped compounds in the literature reveals a striking trend (Fig. 3c). As one might expect, the reported tilting of the CuO_6_ octahedra is inversely proportional to the mean size of the *A* site cation, |*A*|. Plotting *φ* from our single-phase, LTT refinement of the LESCO single-crystal data shows the value to sit within the bounds of the linear relationship that arises from the literature. However, the LTT and LTO components from our two-phase model have *φ* significantly above and below this line, respectively. A similar phenomenon is observed for LBCO and, while the LTO component of our dual-phase model maps to the aforementioned trend, *φ*_LTT_ is clearly greater than previously realised. We attribute the closer fit of our single-phase LTT pXRD models to the true, dual-phase values to a better resolution of key superstructure intensities in the modern, high-resolution pXRD data.

The observation that there appears in the LTT phase a critical tilt angle for the CuO_6_ octahedra, *φ*_c_ ~ 3.6°, above which bulk superconductivity is suppressed^45^ has been well adopted in the discussion of the lanthanum cuprates^5^ (and references therein). In this context, it has also been accepted that the LTO → LTT phase transition acts to change only the direction and sense of octahedral tilting, otherwise resulting in a negligible perturbation to the magnitude of *φ*^18^. The tilt angle in the LTT phase has typically then been extrapolated from the orthorhombicity of the LTO phase (*i.e.*, the deviation of the crystallographic *a* and *b* parameters from equivalence) at *T*_LTT_ and assumed to be invariant with further cooling^48^. Our data for LBCO and LESCO provide direct interrogation of the evolution of this and associated parameters and show clear and physically significant differences to these past assertions.

We find that the phase transition in LESCO affects a significantly greater distortion in the resulting LTT phase compared to that of the LTO phase. Coupled with the altered propagation vector, this results in a much greater buckling of the bent Cu–O–Cu bond in the LTT phase than in LTO [*θ* = 11–12° *vs*. ~ 4°, respectively] (Fig. 3b). The case for LBCO is much the same and the buckling of these interactions in the two phases remains distinct in magnitude from one another, albeit to a lesser degree, with greater buckling of Cu–O–Cu for the LTT component. A similar phenomenon is observed in our recent study of the plain band insulator and structural surrogate, La_2_MgO_4_^21^. We find a discontinuous jump across the phase transition in the octahedral tilt magnitude (captured by *θ* and *φ*) as the phase coexistence evolves to ultimately exclude the LTO phase. Via DFT calculations, we show that this occurs because the LTT phase can accommodate more extreme tilts and buckles than LTO can from a ground state energy perspective. Furthermore, the lack of phase coexistence in that system strongly supports an argument that the phase segregation observed here in doped lanthanum cuprates is of electronic rather than structural origins.

While the limited compositions studied herein do not allow us to evaluate the accuracy of the value provided in the literature for *φ*_c_, it is evident that the assumptions underpinning much of the data from which it is derived are perhaps not ubiquitously valid. Nevertheless, on the basis of our precise refinements of the LTO phases in LBCO, LSCO and LESCO, which span a significant range of |*A*|, it is tempting to suggest that *φ*_LTO_ evolves to a maximum that is largely invariant with |*A*| for the 1/8th-doped lanthanum cuprates, adopting a value of ~ 3.5°, similar to that otherwise inferred for *φ*_c_. In contrast, the LTT phase seems readily able to accommodate the larger distortions favoured by smaller |*A*|. This is further consistent with our study of the structural surrogate, La_2_MgO_4_^21^.

It should be noted here that *φ*_LTO_ appearing to reach a maximum of 3.5°, in the order of *φ*_c_, should not be taken to imply equivalent tilting in the LTO and LTT phases as having equivalent consequence on the electronic band structure of these materials. Even if tilt magnitudes were conserved across the phase transition, there would be a significant difference in the buckling of Cu–O–Cu in the CuO_2_ planes: we extrapolate from our models that a value of 3.6° for *φ* results in those bonds being buckled by approximately 4 and 7° for the LTO and LTT phases, respectively (See Supplemental Fig. 13 and consider Fig. 1e,f). In a case where the Cu 3*d*_*x*_^2^ −_* y*_^2^ and O 2*p*_*x*,*y*_ orbitals are prominently implicated in conductivity^49^, it would seem reasonable to suggest that the Cu–O–Cu bond bending may instead be the parameter of importance, rather than the simple canting of the copper oxide octahedra. With this in mind, the significantly greater buckling of the CuO_2_ planes in the LTT compared to LTO phase of LESCO may in itself lead to significant band narrowing and hence favour CDW formation.

Given the apparent linear dependence of *φ* with |*A*|, on first consideration it is surprising that LSCO, lying somewhere between LBCO and LESCO in Fig. 3c, does not exhibit the same coexistence of phases at low temperature, despite a greater *φ* being expected than that in LBCO. One probable explanation for this anomalous behaviour can be found in the Attfield variance effect^50,51^. Here, an increase in the *A*-site size variance, which would presumably correlate to an increased variation of *φ*, has been correlated with lowering the critical temperature of various properties in similar systems. In this context, LSCO has significantly lower variance in |*A*| than all other systems reported in Fig. 3c [1.1 × 10^−4^ *vs*. 1.8–3.2 × 10^−4^ Å^2^ for LESCO and La_1.875−*y*_Nd_y_Sr_0.125_CuO_4_ (*y* = 0.2–0.8), and 8.3 × 10^−4^ Å^2^ for LBCO]. This cannot be used to directly explain the null observation of LTT, however, for this 1/8th doping and indeed others across this family of compounds, SC persists to the highest temperature in LSCO, a fact that can be attributed to this variance effect, *i.e.*, *T*_c_ is optimised at the lowest *A*-site variance^50^. If you then adopt the view that SC is confined to the LTO phase and imparts an additional electronic stabilisation to its free energy, the suppression of the intrinsic phase coexistence we otherwise observe in LBCO and LESCO can be explained. Further work is clearly needed to elucidate the coupling between electronic instabilities and intrinsic phase segregation in these systems.

A similar intrinsic phase segregation is seen in the colossal magneto resistant (CMR) manganites, wherein a coexistence of the ferromagnetic (FM) and insulating antiferromagnetic (AFM) phases is observed at critical doping^52^. Indeed, we have recently shown, by using a prototype system with a greatly reduced *A*-site variance, that it is possible to suppress this competition all together^53^. While the sense/magnitude of the octahedral rotations in the intrinsically phase-segregated CMR manganites appears to remain beyond current experimental reach, the clear interplay between octahedral rotations, band-narrowing and metal-to-insulator transitions means they are likely to be distinct in the different phases, *i.e.*, electronic and lattice degrees of freedom are intrinsically coupled. As discussed herein, our data finds the magnitude of the octahedral tilts to be distinctly different in the LTT and LTO phases of the cuprate LTO+LTT system. Specifically, resolving the phase-coexistence in the low-temperature data has revealed that the magnitude of the tilt in the LTT phase is larger than previously realised. In this context, the LTT phase would perceivably experience a greater degree of band-narrowing along certain lattice directions and so providing a route by which to stabilise charge-ordering and the suppression of superconductivity.

Considering that the anomalous suppression of SC at *x* = 0.125 is not only seen in La_2−*x*_Ba_*x*_CuO_4_^6^ and La_1.8−*x*_Eu_0.2_Sr_*x*_CuO_4_^11^ but in alternatively-doped systems such as La_1.6−*x*_Nd_0.4_Sr_*x*_CuO_4_^9^, these results appear to call for a revisitation also of the structural analysis of the systems residing around LESCO in Fig. 3c. Moreover, there exist similar trends in the correlation of *φ* with the degree of doping and |*A*| at other values of, and indeed spanning, doping in *x*^18,54^. This appears to extend the potential ramifications of our study beyond just the 1/8th doping regime and implies that such phase segregation may be far more prevalent than thus far realised. Indeed, this is an expectation outlined in recent computational work^49^, the first of its kind to employ functionals that successfully account for various electronic properties in these systems.

## Conclusion

We confidently find by single crystal and powder XRD measurements that, in LESCO at the *x* = 1/8th doping level, an intrinsic phase segregation between LTO and LTT domains occurs down to 10 K. Our PXRD results for LBCO match our findings for LESCO and thus reinstate the discussion of intrinsic phase coexistence in the cuprates for a broad range of average *A*-site ionic radii. This work calls for a revisitation of studies into the interplay of CDW and SC in particularly LESCO which has hitherto been treated as purely LTT at low temperature. Our robust modelling of these two phases against both scXRD and pXRD data reveal a substantial increase in the magnitude of the octahedral tilts in the LTT compared to LTO components therein. Our results strongly suggest that tilting angles in the literature have been incorrectly evaluated on the basis of single-phase refinements and that, at the 1/8th doping regime, many systems may be a phase coexistence between LTO domains of octahedral tilting, *φ*, ~ 3.5° and LTT domains with *φ* strongly dependent on the ionic radii of the *A*-site. We point out that structural differences between the phases are even more stark when one considers the more physically significant buckling of the CuO_2_ planes. These differences should have a substantial effect on electronic structure in LTT, suggesting an explanation as to why superconductivity is inhibited and the CDW instability is instead favoured. Finally, we suggest that the null observation of phase coexistence in LSCO at *x* = 1/8th doping may be linked to low *A*-site variance serving to stabilise the LTO phase through the presence of pronounced superconducting correlations that are reported for this composition.

## Method

### Sample preparation

Polycrystalline La_1.875_Ba_0.125_CuO_4_ was synthesised by a solid-state reaction. Stoichiometric amounts of pre-dried La_2_O_3_ (99.99%, Sigma Aldrich), BaCO_3_ (99.95%, Alfa Aesar), and CuO (99.999%, Sigma Aldrich) were ground into a homogenous mixture, pressed into pellets and calcined in air at 900 °C for 20 h. Further heating cycles at 1050 °C for 15 h, 1100 °C for 20 h and 1200 °C for 20 h with intervening grinding and pelleting produced a high purity sample, as evidenced by the quality of the diffraction pattern.

For LESCO and LBCO, polycrystalline feed rods were first synthesised using stoichiometric amounts of pre-dried La_2_O_3_ (99.999%), Eu_2_O_3_ (99.996%) and SrCO_3_ (99.994%) with 1% excess CuO (99.995%), all sourced from Alfa Aesar. Samples were sintered first at 950 °C for 14 h before five subsequent sintering stages of heating at 1015 °C for 12 h, between each of which the sample was manually ground and subsequently ball-milled at 600 rpm for 30 min. Rods were cast in alumina tubes, compacted by vibration and heated at 980 °C (LSCO) and 995 °C (LESCO) for 12 h, removed from the mould and further heated at 1200 °C for 6 h. Single crystal samples of LESCO and LSCO were prepared using the travelling floating zone furnace method and oxygen stoichiometry was restored by further annealing of the single-crystal samples under flowing O_2_ gas at 800 °C for 2 weeks. Slices and subsequently chips were taken from the much larger single-crystal for diffraction experiments; for the powder studies, chips from those same large samples were ground.

### Single-crystal X-ray diffraction

Single-crystal crystal chips of dimensions 0.12 × 0.05 × 0.02 mm and 0.06 × 0.04 × 0.02 mm of LESCO and LSCO, respectively, were mounted using a 1:1 *v*/*v* mixture of light mineral oil and Parabar 10,312 onto extruded borosilicate capillaries (diameter ≈ 40 μm). Data were collected on a Rigaku Agilent Super Nova II diffractometer using mirror-monochromated Mo *K*α radiation (λ = 0.71073 Å), generated by a micro-focus sealed-tube source, detected at an Atlas 2 CCD detector. Temperatures were controlled using He (30, 70 and 100 K for LESCO, 30 and 50 K for LSCO) and N_2_ (at 130, 150 and 200 K for LESCO, 120 and 150 K for LSCO) gas by an Oxford Cryosystems N-Helix which was operated at a lowered position with the samples surrounded by a beryllium shroud. Further data collections were made at 100 and 150 K for LSCO under N_2_ and without the Be shroud in place.

Indexing, data reduction and initial refinalisation were performed using *CrysAlisPRO* with a numerical absorption correction based on Gaussian integration over a multifaceted crystal model^55^. In the case of data reduction, care was taken to ensure that integration boxes were sufficiently large as to simultaneously encompass all components (*i.e.*, reflections owed to the LTT phase and those owed to each of the two twin components of the LTO phase) and no rejection conditions were applied. Outliers in the primary data were flagged by equivalence comparison and subsequently merged using *SORTAV*^56^ to reduce contamination by multiple scattering events and partial or overlapped reflections. Select reflections were further omitted during the final stages of refinement following their identification as extreme outliers with respect to *F*_obs_/*F*_calc_. Experimental crystallographic tables for the final reported models can be found in the supplementary information. The structure was initially solved using *SHELXS*^57^ and subsequent temperatures by isomorphous replacement, being further refined using *SHELXL17*^58^ implemented through *Olex2*^59^.

To allow an unbiased assessment of the reflection statistics, structural models were again built in *Pccn* in the standard setting such that *a* < *b* and with a twin law, ([0 −1 0], [1 0 0], [0 0 1]), implemented to account for the expected microtwinning in the orthorhombic phases. All reflections are indexed with respect to this setting. Appropriate constraints were imposed upon the atomic positions to raise the symmetry as necessary, with the *A*-site component metals of a given phase constrained to their nominal occupancy and to a single position. This allowed for the concurrent, competitive refinement of structures of differing symmetries, forming models which described two phases in coexistence. Atomic displacement parameters (ADPs) for the *A*-sites were constrained to their appropriate site symmetries with a similarity restraint applied across the components of the two-phase models; atoms in the CuO_2_ planes were constrained to 4/*mmm* point-group symmetry and all O atoms therein constrained to be equal; apical O sites were refined to be isotropic and restrained to be similar between phases for the dual-phase models. An additional, global restraint towards isotropicity of ADPs was required for data of LSCO collected at 100 and 150 K without the Be shroud. While a range of constraints were tested in establishing dual- *vs.* single phase models, this degree of complexity and constraint was optimal in obtaining a set of self-consistently, physically sensible, fully converged refinements for the final models.

We take as a better estimate of the true lattice parameters for the purpose of geometric data in LESCO those derived from the dual-phase Rietveld refinements which are unbiased by the process of modelling multiple domains with a single orientation matrix. Conventional single-crystal CIFs are nevertheless also deposited.

### Powder X-ray diffraction data collection and refinement

Variable-temperature (VT) powder X-ray diffraction (pXRD) data were collected on samples of LESCO and LBCO at beamline 09A and for LSCO at beamline 19A of the Taiwan Photon Source, all three using radiation of approximately 20 keV with a MYTHEN detector (the precise value for each experiment being determined by refinement against a LaB_6_ standard). For the low temperature data of LESCO and LBCO, temperature was controlled using an ESRF DynaFlow cryostat and collected at 10 K intervals upon heating from 10 to 130 K and 10 to 140 K for LBCO and LESCO, respectively, at a rate of ~ 1.67 K/min, while LSCO was scanned at 5 K intervals between 15 and 110 K; LSCO data at 105 K exhibited a systematic error and is omitted. For collections on LESCO, the sample was sealed in a Lindemann glass capillary of diameter 0.3 mm, while the sample of LBCO was sealed in a 0.2 mm diameter quartz capillary. Both samples were rotated during data collection to improve powder averaging. Further data for LESCO were obtained at 10 K intervals from 150 to 200 K using the same beamline and detector but with radiation of approximately 12 keV in energy and temperature controlled using an Oxford Cryosystems Cryostream 800+ , heating at a rate of ~ 2.5 K/min.

Rietveld refinements against the diffraction patterns were performed using *TOPAS-*Academic (version 6)^60^*,* first for the LESCO sample, where results were benchmarked against single crystal refinements. The resulting, optimised Rietveld refinement procedure then allowed for an informed route to be undertaken to correctly fit the data for LBCO. All refinements were carried out in *Pccn*, the highest common subgroup of the LTO (*Bmab*) and LTT (*P*4_2_/*ncm*) phases, with appropriate constraints used to ultimately describe the higher symmetry models as necessary. The background at low temperature was modelled using 18 terms of a shifted Chebyshev polynomial series and careful implementation of 9 gaussians for LESCO and LBCO, 8 for LSCO, to account for broader features owed to the sample environment. For LESCO data between 150 and 200 K, 6 terms of the shifted Chebyshev polynomial series and 3 gaussians were employed. A better estimation of absorption was taken by its refinement and averaging over the temperature range in the context of imported values for atomic displacements (first from the single crystal for LESCO and LSCO, and from LESCO for LBCO) at a range of temperatures.

The final refinements proceeded with atomic positions refined using the symmetry-adapted displacement formalism^61^, implemented through the web-based software ISODISTORT (version 6.10.0)^62,63^. For all phases, individual components of the X_3_^+^ and Γ_1_^+^ modes were constrained to refine as functions of a single common parameter for each mode with the specific ratios used being extrapolated from the single-crystal data, using those of LESCO for refinements of LBCO. For LESCO and LBCO, isotropic displacements (*B*_eq_) were refined for each atom site type and, in the LTO+LTT model, constrained to be equal across phases, while a further similarity constraint was ubiquitously applied to *B*_eq_ for oxygen atoms: equatorial sites were set to be equal, regardless of phase symmetry, with apical sites refined to be 1.8 times greater, again informed by single-crystal data. In the case of LSCO, it was necessary to further constrain the *A* and *B* site *B*_eq_ to be equal. Anisotropic Stephens^64^ models of strain were employed for each phase in all samples, with LTO+LTT models employing a constrained interpolation of the LTT ζ parameter (vide infra). For LESCO only, a single Gaussian function relating to domain-size dependent peak broadening was also refined. Data for LSCO revealed a complex strain system that was best modelled by implementing two LTO phases of identical cell, atomic position and displacement parameters but allowing each a separate set of Stephens broadening parameters.

Extensive testing of differing model complexities was performed for the LESCO sample to reach the above model, paying close attention to the physical plausibility of parameters and their trends with temperature as well as their impact on the fit. By constraining component modes within the X_3_^+^ and Γ_1_^+^ sets to refine as functions of a single value, stable refinement of light atom positions could be obtained and resulting geometries matched well to those of the single-crystal data. Free refinement of these parameters would instead result in occasional mismatch of sign (*i.e.*, direction) for individual components that could otherwise not be stabilised into physically reasonable minima, as well as resulting in less steady geometric trends. Such treatment had otherwise minimal impact on the results. We also note a correlation between strain parameters and phase fraction. Here, correlations with the ζ Stephens parameters, defining the Gaussian and Lorentzian contributions to the peak shape, intermittently destabilised refinement of cell parameters and the phase fraction towards clearly false minima, so producing unreasonable trends. Analysis of their behaviour across the temperature regime for a range of models allowed for a physically plausible extrapolation of this parameter in the LTT component of the LTO+LTT model, being a maximum at 10 K and steadily trending to 0 (purely gaussian) at the onset of the phase transition to a purely LTO phase. This work, in comparison with the single crystal data for LESCO, enabled an abridged approach to be taken for LBCO once similar problems were identified. In the case of LSCO, intensity for key superstructure peaks was sufficiently weak and obfuscated that reasonable and meaningful geometric data were unobtainable and are hence omitted from discussion.

### Crystallographic phase analysis

Single-crystal X-ray diffraction allows for an analysis of individual symmetry components through the assessment of the adherence to certain reflection conditions. With reference to the low-temperature supercell and in the context of *a*-*b* twinning of the *Bmab* (LTO) phase, these can be divided into three categories. First and of least concern, reflections obeying the aristotypical diffraction condition, *h* + *k*, *h* + *l*, *k* + *l* = even (F_HTT_), which are expected to be insensitive to the identity of any given phase as they contain no information about the sense or magnitude of the distortions transforming as the irreducible representation X_3_^+^ of *I*4/*mmm*. Second, the subset of super-structure reflections given by *h* + *l*, *k* + *l* = odd (SS_LTT_) which correspond to violation of the *B*-centring condition in a twinned system. These arise from rotation of the X_3_^+^ propagation vector away from (a;0). Observation of SS_LTT_ can, therefore, be considered as direct corroboration of the presence, or else absence, of the *P*-centred LTT (*P*4_2_/*ncm*) or LTLO (*Pccn*) phases. Finally, the remaining allowed violations of the aristotypical diffraction condition, designated SS_common_, correspond to super-structure reflections common to both LTT and LTO (and LTLO) phases. These are expected to be sensitive to the presence of an orthorhombic phase and associated twinning, since they arise exclusively from the X_3_^+^ distortions. In the case of a tetragonal system, these would follow F_*hkl*_ = F_*khl*_, a condition that is inherently broken for a single domain orthorhombic phase, and even in the cases of a near 50% twining of the LTO domain, by virtue of the fact that F_*hkl*_^2^ + F_*khl*_^2^ ≠ (F_*hkl*_ + F_*hkl*_)^2^. Thus, with the careful measurements of the weak superstructure peaks performed here, it is still possible to discriminate between not only the case of LTT and LTO single phases but also possible LTT+LTO mixtures. Supplemental Fig. 1 reports the calculated *R*-factors for *F* (*R*_1_) for these three reflection classes, as well as *R*-factors output by *SHELX* for the complete data, for the various models discussed.

It is pertinent at this stage to acknowledge an associated challenge of modelling phase coexistence in single-crystal data within the confines of the *SHELXL* software: it must be performed as disorder model. In this context, the model assumes that the phases coexist within the coherence length of the experiment and, hence, [F_obs_^2^(LTT) + F_obs_^2^(LTO)] ≡ [F_obs_(LTT) + F_obs_(LTO)]^2^. While this is clearly not strictly true, for the stronger reflections that are allowed in both components this equivalence is found to hold and, more generally, the phases of the structure factors are almost entirely conserved. This equivalence breaks down most in the refinement where contributions from the LTO phase are systematically absent and, as a consequence, the super-structure peaks owed to solely the LTT phase are underestimated. This is expected to amplify the absolute magnitude of the X_3_^+^ distortion therein. However, since we find good agreement with the powder diffraction data which appropriately treats these components as distinctly separate, we conclude the discussion to be valid and this systematic error to be largely overcome by information from those more numerous and more intense superstructure reflections.

At 150 K, our single crystal study finds clear adherence to the extinction conditions associated to the twined LTO phase, with no significant intensities observed for SS_LTT_. Below this temperature, there are violations of the *B*-centring extinction condition as the phase composition changes, falling rapidly to 58.1(18)% LTO at 100 K and reaching 45.1(2.7)% at 30 K for the LTO+LTT model. Figure 2b presents the impact of the main refinement models discussed herein to the *SHLEX R*_1_ discrepancy index for the single-crystal study of LESCO, while a more comprehensive report of fitting statistics, including for individual reflection subsets, may be found in Supplemental Fig. 1. The data unequivocally discriminates against a single-phase *P*4_2_/*ncm* (LTT) model at all temperatures. This is illustrated best by considering the fitting statistics for SS_common_ which are appreciably higher for the purely tetragonal system than for any orthorhombic-containing model. While these are largely inconclusive with respect to the occurrence of a single-phase LTLO model, as opposed to a coexistence of two phases, this can be discounted based upon analysis of the powder data (vide infra). It follows then that the single-crystal reflection statistics are unable to discriminate between the LTO+LTT model and those in which the LTO or LTT component is replaced by one of LTLO form (LTLO+LTT and LTO+LTLO, respectively). This is unsurprising since the structure factor of LTLO will refine to vary continuously between that of LTT and LTO. In general, the SS_LTT_ reflections were modelled better by increasing parameterisation of the refinement (*i.e.*, LTO+LTT < LTO+LTLO < LTLO+LTT), but this trend was less well observed in SS_common_. Moreover, analysis of the atomic positions across the various dual-phase models showed no consistent, significant departure of CuO_6_ octahedra from their higher symmetry axes in the LTLO components with respect to the LTT or LTO components that they replace (Supplemental Table 2). This lacking impact of the additional degrees of freedom provided by the inclusion of an LTLO component in the refinement supports selection of the equally well-fitting, more sparsely parameterised LTO+LTT description. As expected, F_HTT_ showed only slight bias between the models and in no consistent manner.

In comparison to a single-phase LTT model, LTO+LTT reduced the *R*_wp_ for powder diffraction data of LESCO by ~ 1.2% [*ca*. 2.2 *vs*. 3.4%] (Fig. 2c) below 100 K and provided a level trend for the fitting statistic across the phase transition regime. This corresponds to a rapid change from pure LTO at 150 K to comprising 54.73(15)% LTO at 120 K and reaching 44.1(2)% at 10 K. In comparison, refinement of a single-phase LTLO model provided only a small and inconsistent improvement against the LTT model below *T*_LTT_ [∆*R*_wp_ < 0.15%]. No meaningful changes in the structure or quality of the fit were observed in replacing either LTT or LTO component of the dual-phase model with an LTLO phase. This lacking distinction is to be expected since the number of well resolved super-structure reflections, themselves necessarily powder-averaged across pseudo symmetry equivalents, is rather small [~ 15] and all are generally very weak. The implementation of a dual-phase model prominently improved the visible fit where an orthorhombic distortion would affect peak shape and to those where the differing *B*- and *P*-centring extinction conditions are present (Fig. 2e–h).

While the single crystal data is superior in probing the systematics and intensities of the weak SS peaks, it fails to resolve whether the deviation from pure-LTT arises from phase segregation or a single phase of intermediate geometry (*i.e.*, a single LTLO phase). The strength of the high-resolution powder diffraction data is in the ability to precisely interrogate peak profiles. In this context, splitting and asymmetry of the strong *h* + *k*, *h* + *l*, *k* + *l* = even, aristotypical reflections may only be satisfactorily accounted for by a two-phase model with orthorhombic and tetragonal lattice symmetries. Thus, through the combination of the two experiments, we confidently assign the temperature-dependent structural behaviour of LESCO as comprising a distinct coexistence of the LTO and LTT phases below *T*_LTT_ to at least 10 K.

The high degree of consistency between single-crystal and powder data for LESCO, each providing different handles by which to interrogate the LTT and LTO phase coexistence, gives us confidence in interrogating our low temperature pXRD LBCO results. Compared to refinement of a single LTT phase, the dual phase LTO+LTT model reduced the *R*_wp_ for the powder diffraction data by around 0.4% [*ca.* 1.45 *vs*. 1.85%] below 50 K with the inclusion of ~ 58% LTO and resulted in a level trend across the transition into the LTO phase (Fig. 2d). Refinement of a single LTLO phase at 10 K returned an *R*_wp_ of 1.66% with the fit growing increasingly worse with heating throughout the LTT regime, *i.e.*, as the LTO fraction increased, and showed no significant benefit compared to a single LTO component at any point. Inclusion of an LTLO component into the dual-phase model also served no improvement on the already described model.

While every effort has been made to ensure the refinement models are as accurate as possible, we note that the calculated uncertainties provided [± 0.75% at 3σ confidence] are not considered as accurately reflecting the true uncertainties in the phase fractions of the LTO+LTT models for the powder data. Specifically, the obtained phase fractions are somewhat more sensitive to choices made in the refinement than the calculated values imply. In this context, based on the results of various modelling efforts, we estimate a +5/−10% margin of error for LESCO (± 4% for LBCO) pXRD data to be more representative in the classically LTT-region and ± 10% (± 8%) during the transition to a purely LTO phase from 120 to 140 K (50–70 K), inclusive. Errors provided by *SHELX* for the scXRD refinements appear to more accurately reflect the sensitivity of the experiment to parameter choice and data handling.

For LSCO, no evidence was found in the single-crystal diffraction data at any temperature to support the existence of any other phase at low temperatures besides the LTO phase: intensities corresponding to violation of the twinned *B*-centring condition represented only noise (Supplemental Figs. 14 and 15). Accordingly, an LTT-type model was a poor fit to the data and, moreover, no stable dual-phase model could be obtained at any temperature. We note that lowering the symmetry to that of the LTLO phase served small improvements to the *R* factors at 30 K (*wR*_2_ = 5.23% *vs.* 5.72% for LTO), corresponding to a slight deviation in the structural geometry and particularly *A*-site thermal parameters towards those of an LTT-type phase, but this is of course unsupported by the previous observation regarding no *B*-centring violations. Compared to a single-phase LTO model, inclusion of a secondary LTT phase in the refinement of the powder diffraction data resulted in an apparently significant improvement to the *R*_wp_ [~ 2.0% *vs*. ~ 2.8%] but we attribute this solely to a more effective modelling of microstrain: the best fits were obtained using two identical LTO phases with the exception of their employing different sets of Stephens broadening functions [*R*_wp_ ~ 1.9%]. We conclude, therefore, that LSCO adopts a purely LTO state at all temperatures studied.

## Supplementary Information

Supplementary Information.

## Data Availability

The powder and single-crystal X-ray diffraction CIFs for the final chosen models, CIFs for those of the single-crystal with unit cell dimensions taken from the powder, CIFs for the rejected, alternate symmetry models for the single-crystal X-ray diffraction data and CIFs corresponding to Supplemental Table 1, as well as powder X-ray diffraction instrument standards, that support the findings of this study are available from figshare, https://figshare.com/projects/Structural_studies_of_LESCO_LBCO_and_LSCO/127661.

## Code availability

All key software and code are publicly or commercially available.
