# Supplementary material for: Pronounced interplay between intrinsic phase-coexistence and octahedral tilt magnitude in hole-doped lanthanum cuprates
Source: Sci Rep. 2022 Aug 22;12:14343. doi: 10.1038/s41598-022-18574-1 (PMC9395519; doi:10.1038/s41598-022-18574-1)
Supplement: Supplementary file 1 — Supplementary Information. [file 41598_2022_18574_MOESM1_ESM.docx]

Pronounced interplay between intrinsic phase-coexistence and octahedral tilt magnitude in hole-doped lanthanum cuprates

Jeremiah P. Tidey,^1^ En-Pei Liu,^2,3^ Yen-Chung Lai,^4^ Yu-Chun Chuang,^4^ Wei-Tin Chen,^3,5^ Lauren J. Cane,^6^ Chris Lester,^6^ Alexander N. D. Petsch,^6^ Anna Herlihy,^1,7^ Arkadiy Simonov,^8^ Stephen M. Hayden,^6^ Mark Senn^1,*^

1. Department of Chemistry, University of Warwick, Gibbet Hill, Coventry, CV4 7AL, United Kingdom
2. Department of Physics, Tamkang University, Tamsui 25137, Taiwan
3. Center for Condensed Matter Sciences and Center of Atomic Initiative for New Materials, National Taiwan University, Taipei 10617, Taiwan
4. National Synchrotron Radiation Research Center, Hsinchu 30076, Taiwan
5. Taiwan Consortium of Emergent Crystalline Materials, Ministry of Science and Technology, Taipei, 10622, Taiwan
6. H.H. Wills Physics Laboratory, University of Bristol, Bristol, BS8 1TL, United Kingdom.
7. ISIS Neutron and Muon Facility, Rutherford Appleton Laboratory, Didcot, OX11 0QX, United Kingdom
8. ETH Zürich, Department of Materials (Multifunctional Ferroic Materials), Vladimir-Prelog-Weg-5/10, 8093, Zürich, Switzerland

* Correspondence to m.senn@warwick.ac.uk


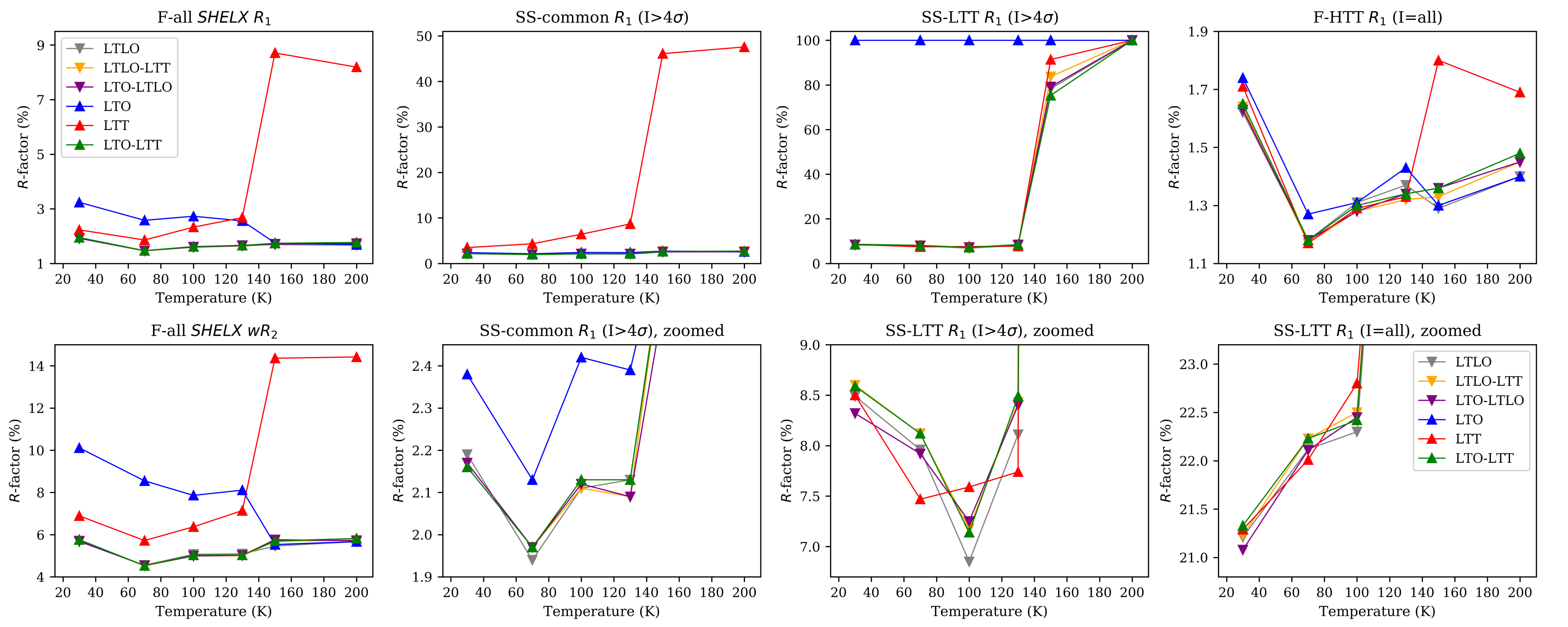


**Figure 1.** Plots of the *SHELX* calculated *R* factors for all allowed reflections and of the calculated *R*_1_ explored for the three classes of reflections – SS_common_, SS_LTT_ and F_HTT_ – as described in Methods section of the main paper and for all differing symmetry single-crystal models tested.


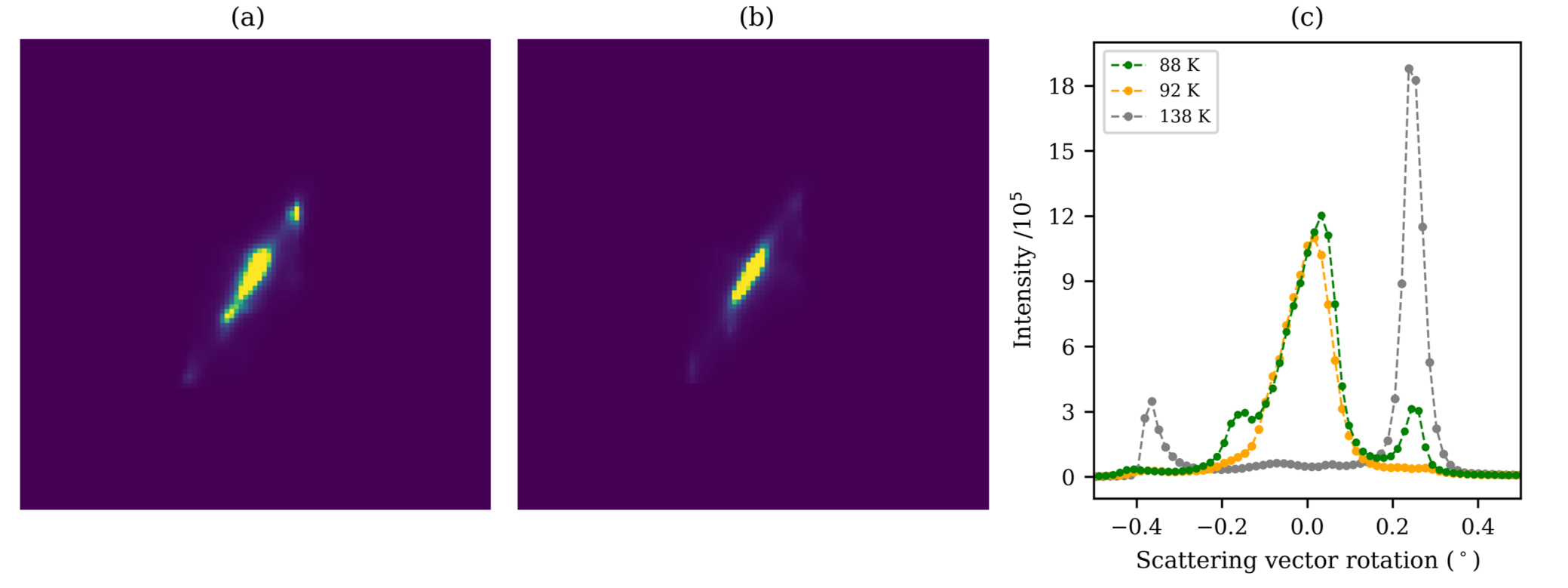


**Figure 2.** (a) and (b) Images of the diffraction from the (7 1 1)/(1 7 1) reflection set at 88 and 92 K, respectively, for a single crystal of LESCO in an experiment designed to resolve those peaks, displaying the central LTT reflection with satellites owed to the twinned LTO phase. (c) Plot comparing the intensity across a line taken through these peaks, with the scattering vector centred at the LTT peak maximum and the LTO peak set at 138 K shown for further reference. Data is collected upon heating using 3 minute scans over a select region of reciprocal space at 100% intensity of the primary beam. Between these experiments, further collections exposed the sample to the full primary beam for 9 minutes and 16 minutes of the beam at 1% intensity. The experiment was performed using EH1 of Beamline I19, Diamond Light Source, where the beam flux is approximately 2.6×10^12^ photons per second.^1^ The data clearly show a coexistence of the LTO and LTT phases at 88 K, while intensity ratios are seen to drop from around 30% LTO to 10% LTO between the experiments, showing the impact that irradiation can have on the phase composition of the system.


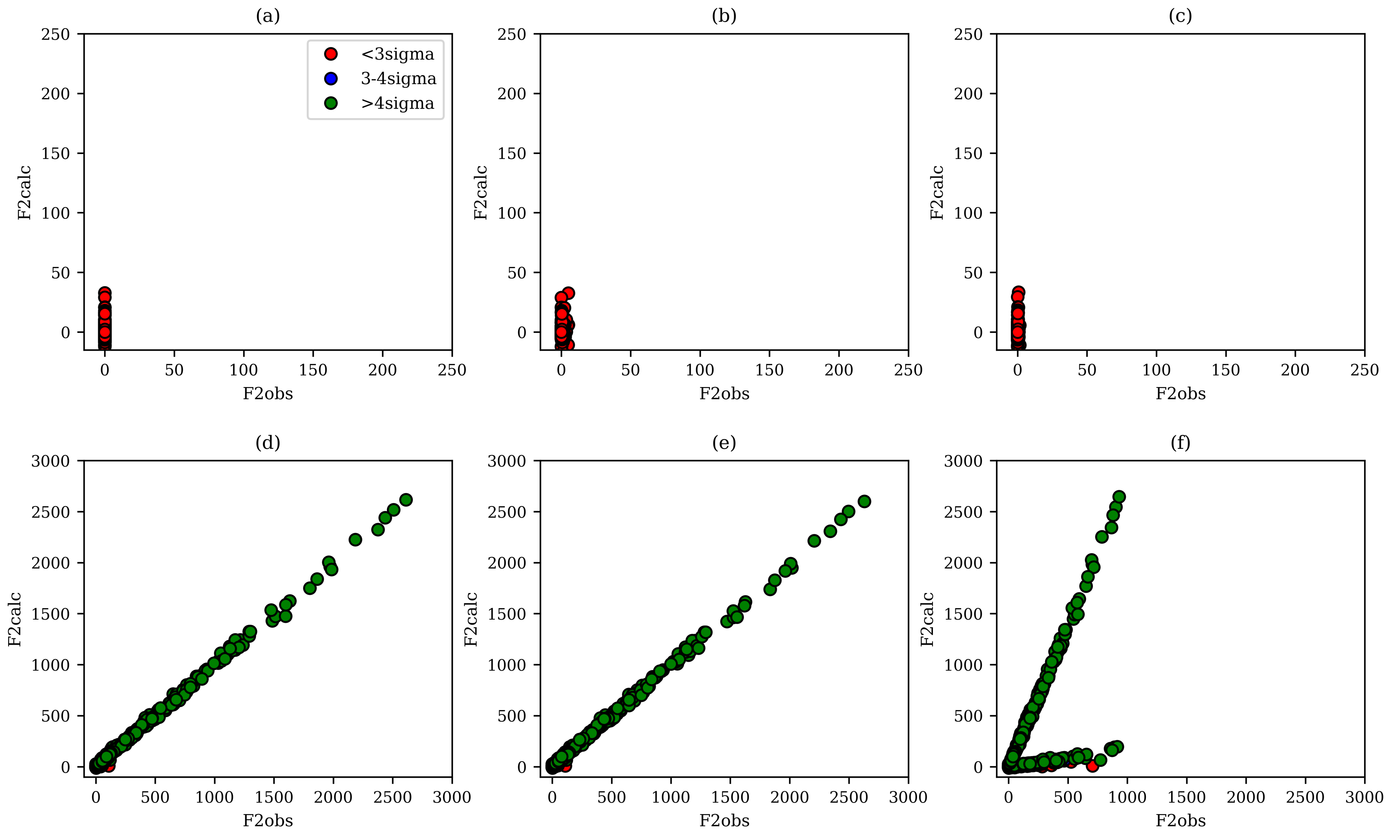


**Figure 3.** Plots of *F*^2^_obs_ vs. *F*^2^_calc_ in the case of the reflection subclasses SS_LTT_ (a,b,c) and SS_common_ (d,e,f) for the LTO (a,d), LTO+LTT (b,e) and LTT (c,f) models of the single crystal data for LESCO at 200 K. Note the lack of intensity to fit in the SS_LTT_ subclass and how the LTT model fails to model the orthorhombic breaking of the *hkl*≡*khl* equivalence.


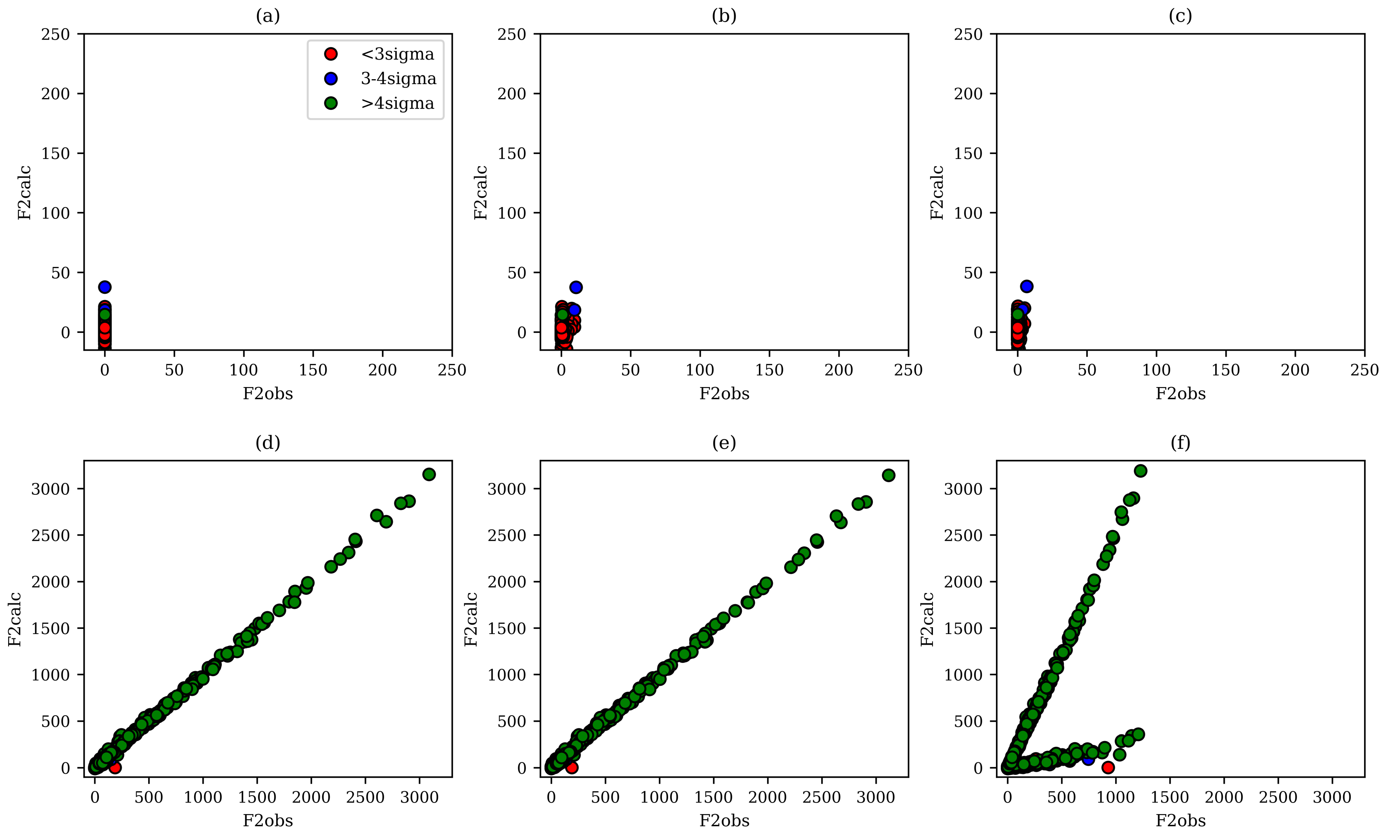


**Figure 4.** Plots of *F*^2^_obs_ vs. *F*^2^_calc_ in the case of the reflection subclasses SS_LTT_ (a,b,c) and SS_common_ (d,e,f) for the LTO (a,d), LTO+LTT (b,e) and LTT (c,f) models of the single crystal data for LESCO at 150 K. Note the lack of intensity to fit in the SS_LTT_ subclass and how the LTT model fails to model the orthorhombic breaking of the *hkl*≡*khl* equivalence.


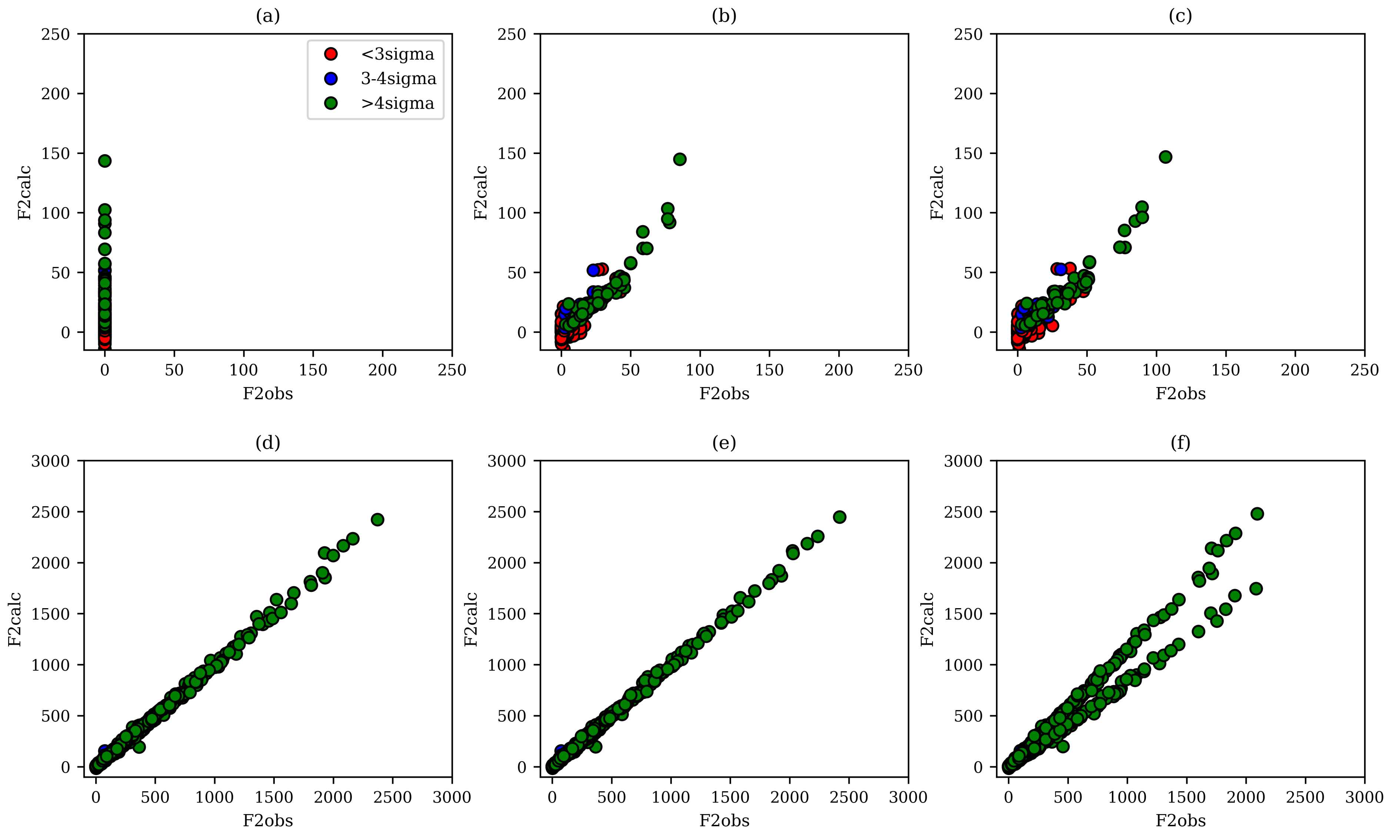


**Figure 5.** Plots of *F*^2^_obs_ vs. *F*^2^_calc_ in the case of the reflection subclasses SS_LTT_ (a,b,c) and SS_common_ (d,e,f) for the LTO (a,d), LTO+LTT (b,e) and LTT (c,f) models of the single crystal data for LESCO at 130 K. Note the LTO-only phase visibly fails to model the SS_LTT_ subclass while the LTT model fails to model the orthorhombic breaking of the *hkl*≡*khl* equivalence.


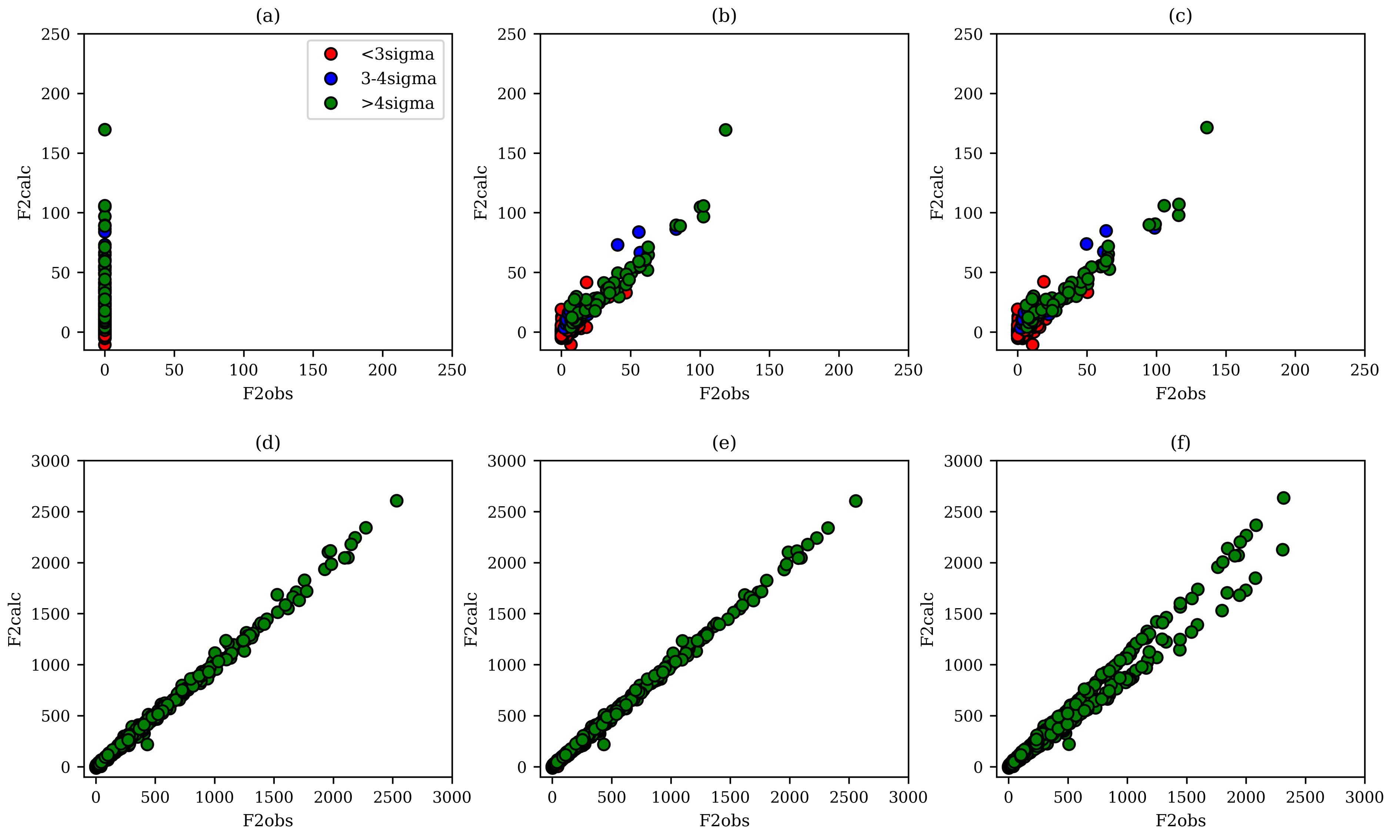


**Figure 6.** Plots of *F*^2^_obs_ vs. *F*^2^_calc_ in the case of the reflection subclasses SS_LTT_ (a,b,c) and SS_common_ (d,e,f) for the LTO (a,d), LTO+LTT (b,e) and LTT (c,f) models of the single crystal data for LESCO at 100 K. Note the LTO-only phase visibly fails to model the SS_LTT_ subclass while the LTT model fails to model the orthorhombic breaking of the *hkl*≡*khl* equivalence.


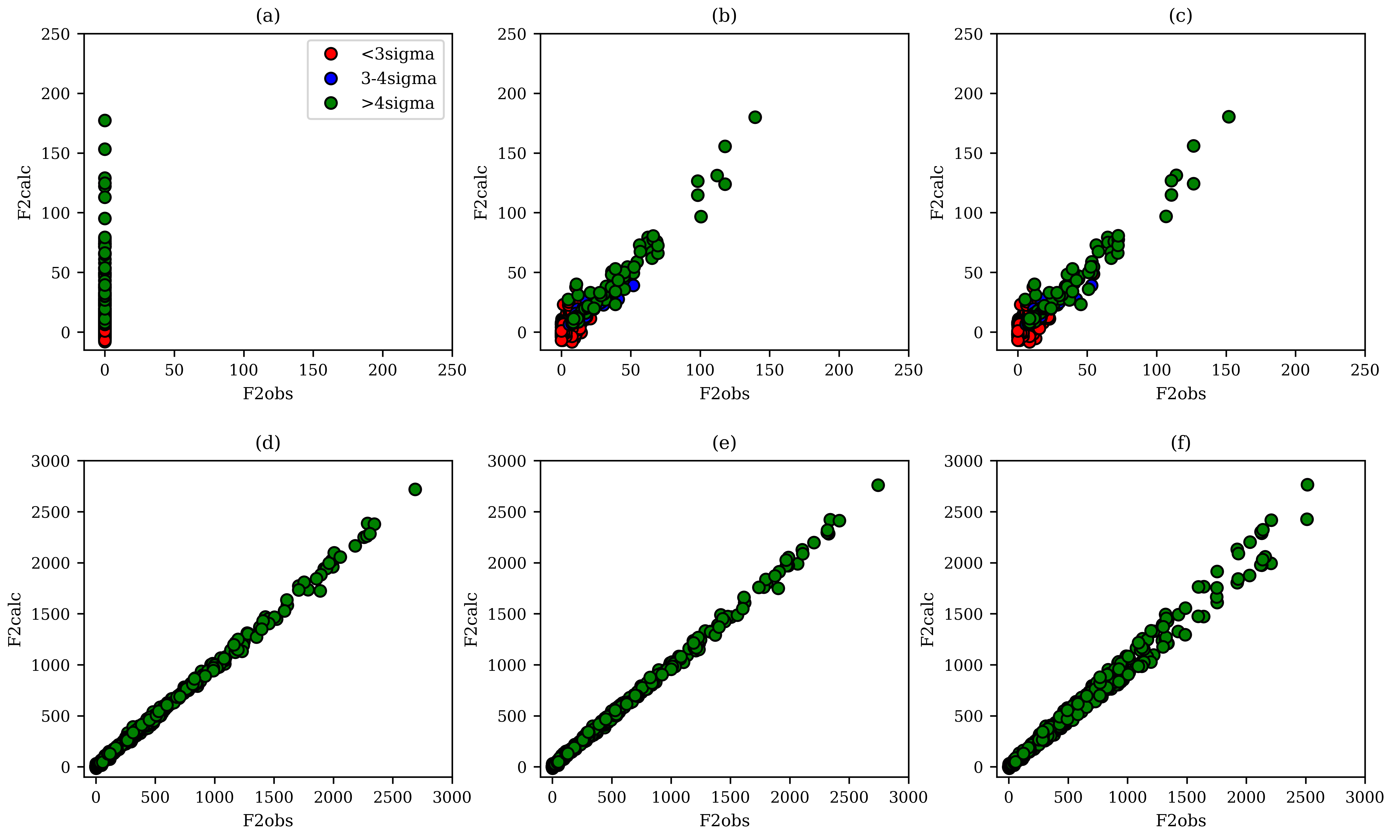


**Figure 7.** Plots of *F*^2^_obs_ vs. *F*^2^_calc_ in the case of the reflection subclasses SS_LTT_ (a,b,c) and SS_common_ (d,e,f) for the LTO (a,d), LTO+LTT (b,e) and LTT (c,f) models of the single crystal data for LESCO at 70 K. Note the LTO-only phase visibly fails to model the SS_LTT_ subclass while the LTT model fails to model the orthorhombic breaking of the *hkl*≡*khl* equivalence.


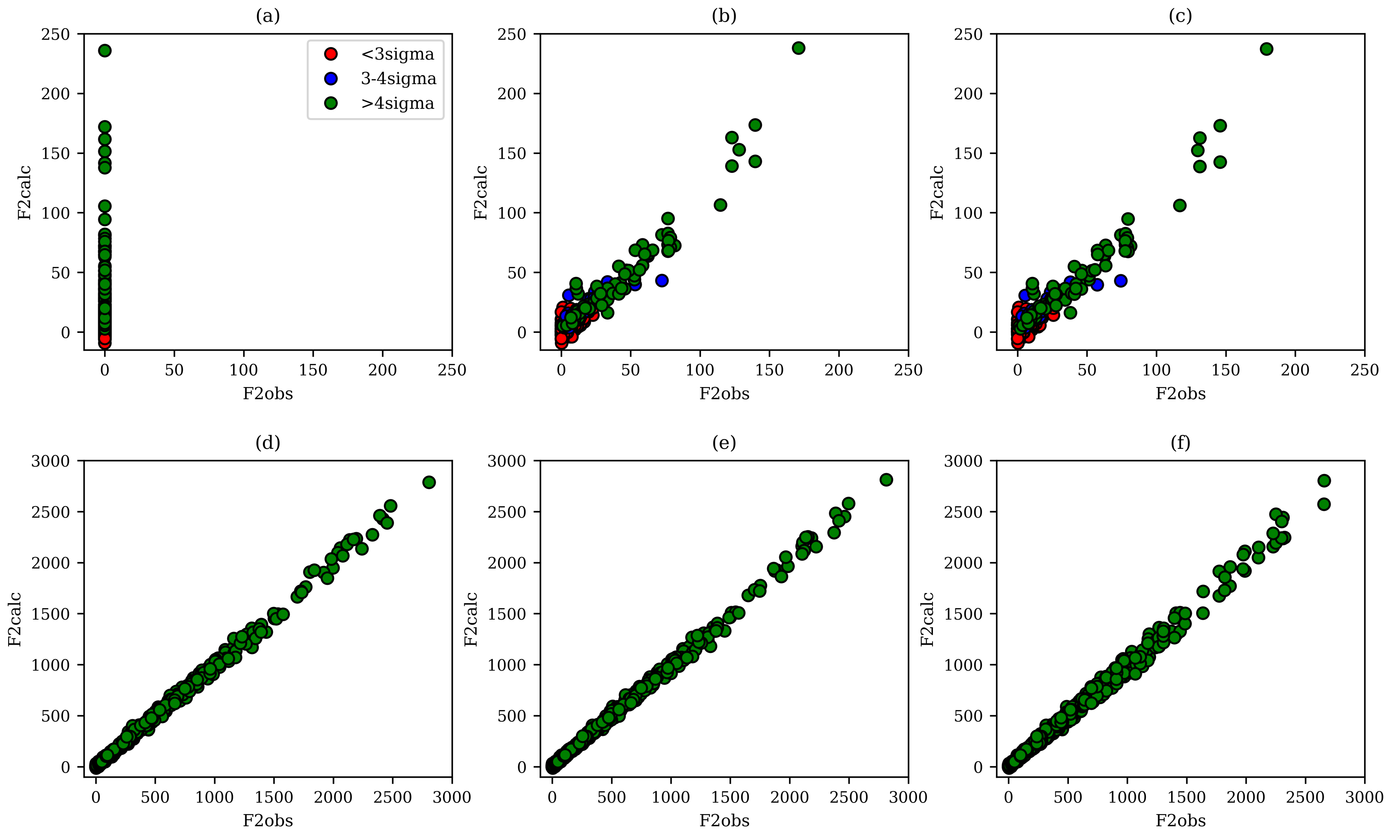


**Figure 8.** Plots of *F*^2^_obs_ vs. *F*^2^_calc_ in the case of the reflection subclasses SS_LTT_ (a,b,c) and SS_common_ (d,e,f) for the LTO (a,d), LTO+LTT (b,e) and LTT (c,f) models of the single crystal data for LESCO at 30 K. Note the LTO-only phase visibly fails to model the SS_LTT_ subclass while the LTT model fails to model the orthorhombic breaking of the *hkl*≡*khl* equivalence.


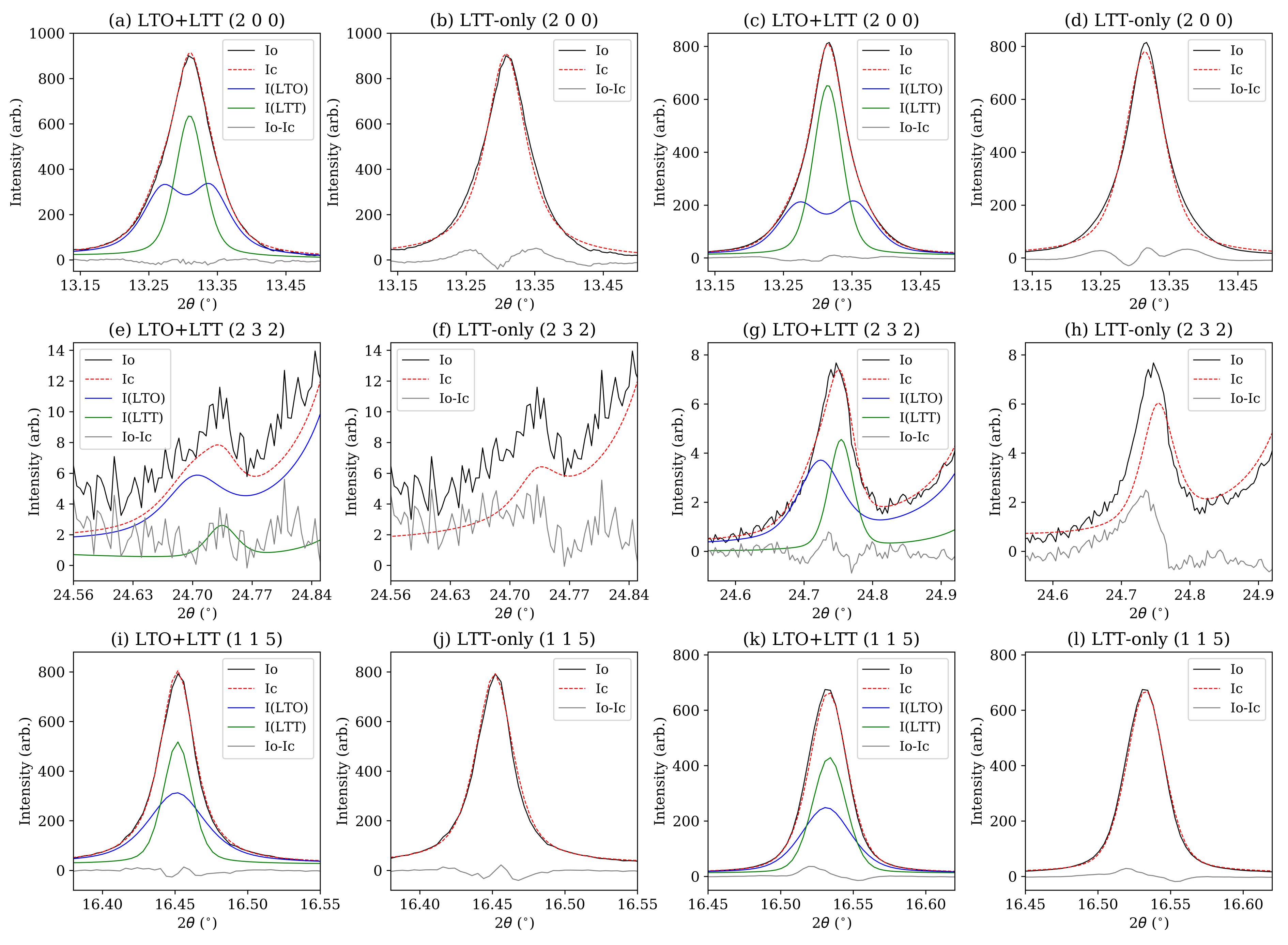


**Figure 9.** Rietveld refinement fit traces of the LTO+LTT and LTT-only models for the (2 0 0)/(0 2 0), (2 3 2)/(3 2 2) and (1 1 5) reflections, as labelled, for LBCO (left two columns, a, b, e, f, i, k) at 10 K and LESCO (right two columns, c, d, g, h, k, l) at 50 K. Note how the refinement model only significantly affects those reflections subject to effects of orthorhombic inequality (top two rows) and not the (1 1 5) reflection.

**Table 1** Phase fractions obtained in LTO+LTT model refinements for data collected below the LTO/LTT phase transformation in a further four different crystals of LESCO. CIFs are deposited in figshare online.

| **Supplemental CIF** | **T (K)** | **R_1_ (%)** | **Crystal dimensions (mm)** | **Composition (%LTO)** |
| --- | --- | --- | --- | --- |
| SI-1_100K | 100 | 2.51 | 0.093 × 0.036 × 0.027 | 65(2) |
| SI-2_100K | 100 | 3.86 | 0.136 × 0.077 × 0.038 | 52(4) |
| SI-3_100K | 100 | 1.93 | 0.124 × 0.077 × 0.01 | 57.7(19) |
| SI-4_95K | 95 | 1.41 | 0.119 × 0.035 × 0.027 | 56.6(16) |

**
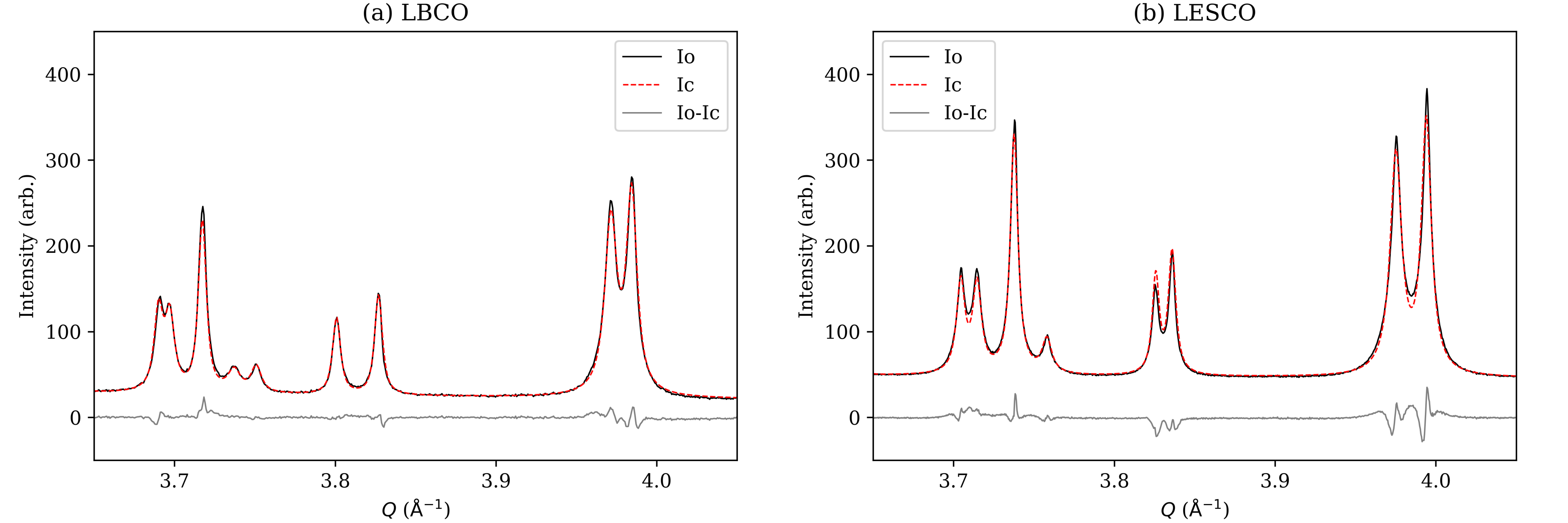
**

**Figure 10.** Characteristic sections of the Rietveld refinement fit traces of the LTO-only model to PXRD data of (a) LBCO and (b) LESCO at 130 and 200 K, respectively, temperatures well within the single-phase regime of each. We attribute deficiencies in these plots to strain, particularly brought about by the mismatch of cell dimensions at domain boundaries, it clearly scaling with ∆(*a*,*b*) and being modelled well with the inclusion of a second, highly strained, LTT-like phase with equivalent *c* parameter (not included in final refinements). Estimated values of e0 are 0.037% for LBCO and 0.034% for LESCO, derived by its refinement in the omission of other strain models and with all other parameters except scale frozen at the reported converged model.


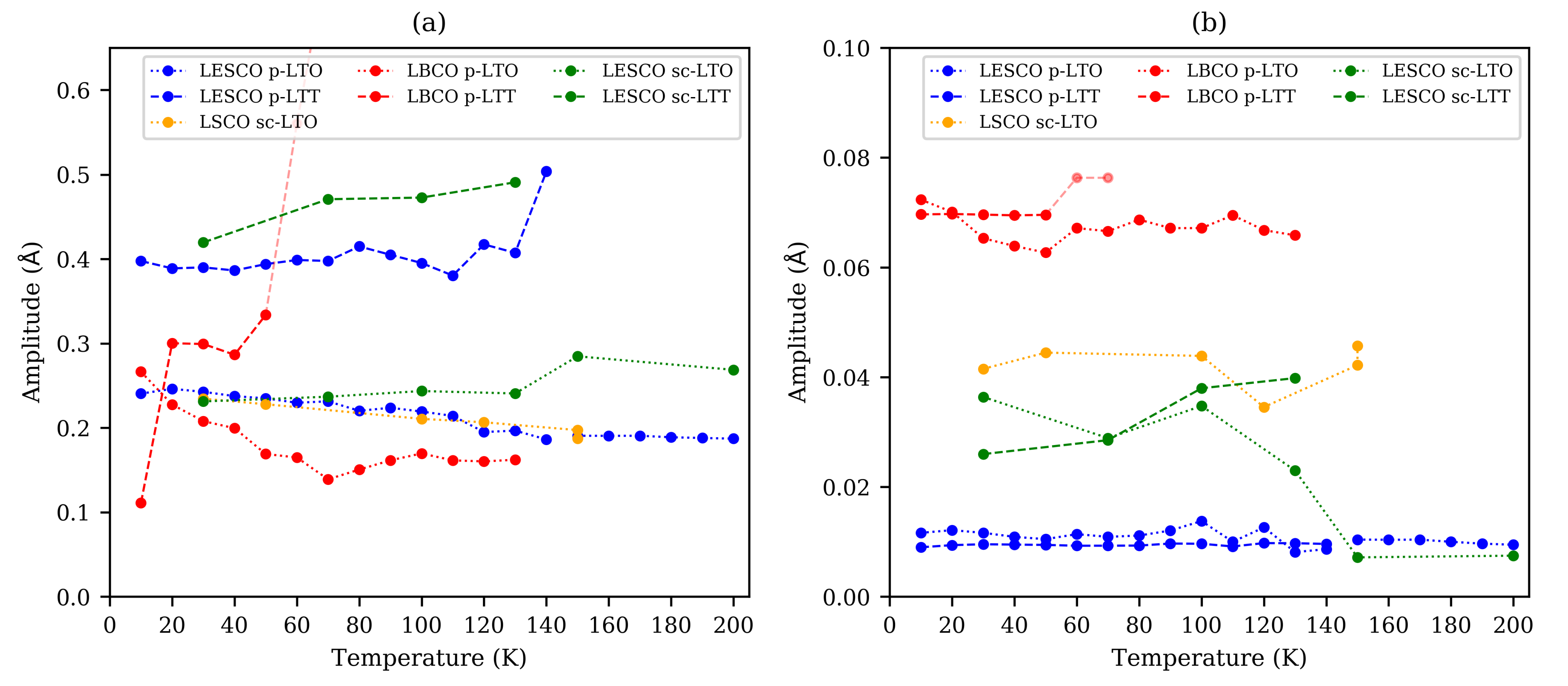


**Figure 11.** Plot of the evolution of the total (a) X_3_^+^ and (b) Γ_1_^+^ mode amplitudes, with *ISODISTORT*^2,3^ used to decompose the atomic coordinates from the powder and single-crystal refinements in terms of symmetry adapted displacements as irreducible representations (Irreps.) of *I*4/*mmm* and implementing the *Ap* normalisation as defined in ISODISTORT. We find the overall total magnitudes of the X_3_^+^ distortions (associated with the octahedral tilting) in LESCO to be 1.6-2.0 times greater in the LTT than LTO components of the LTO+LTT model in both single-crystal and powder diffraction data, while the factor is smaller (~1.4) in the case of LBCO. Only minor evolution with temperature is observed below the phase transition for the and none within the sensitivity of the experiment for the Γ_1_^+^ (*i.e.*, the perturbation of the axial oxygen and *A*-site cations along *c*) modes. This is consistent with a harmonic approximation in which the higher order coupling with X_3_^+^ is taken to be negligible, but we note that the X_3_^+^ thermal dependence is consistently marginally greater in the LTO than LTT phase component. There appears a slight discrepancy between the powder and single-crystal experiments for LTO X_3_^+^ with heating through *T*_LTT_, into the LTO regime. The single-crystal experiment observes an increase of the octahedral tilting, *φ*, with the depletion of the LTT phase [*φ* = 3.42(4) to 4.04(4) ° between 130 and 150 K, respectively] while the powder agrees closely at low temperature but steadily deviates above 70 K to a *φ* of 2.81(4) ° at 150 K. The lack of any dramatic changes in the distortion modes below *T*_LTT_ supports the first order nature of the LTO→LTT phase transition. This is further reflected in LESCO by the observation of relatively temperature-independent cell parameter evolution that instead shows a slight ‘pinching in’ of the *a* and *b* parameters of the LTO phase towards the value of *a* in the LTT phase during the LTT-dominated range (Supplemental Figure 5a). This indicates that the LTO and LTT domains are likely intergrown with each other on a length scale implying strain-fields penetrate into the volume and so affect the coherent diffraction signal. Evolution of cell parameters for LBCO (Supplemental Figure 5) mimic those seen for LESCO across the dual-phase region but transposed to lower temperature: there is negligible change to the LTT-*a* parameter and the indication of a small ‘pinching in’ of LTO *a* and *b* parameters towards those of the LTT phase as temperature is lowered. Across the transitory region (50-80 K), the LTT-*a* appears to coalesce with LTO-*b*, however, the weak intensity and broad peak shape owed to the LTT component at this stage precludes a confident discussion. Just as for LESCO, the unit cell volume of the LTT phase is found to be only around 0.2 Å^3^ smaller than in the LTO phase, although the relative magnitude of the *c* parameters of the phases shows a more substantial difference (Supplemental Figure 5b).


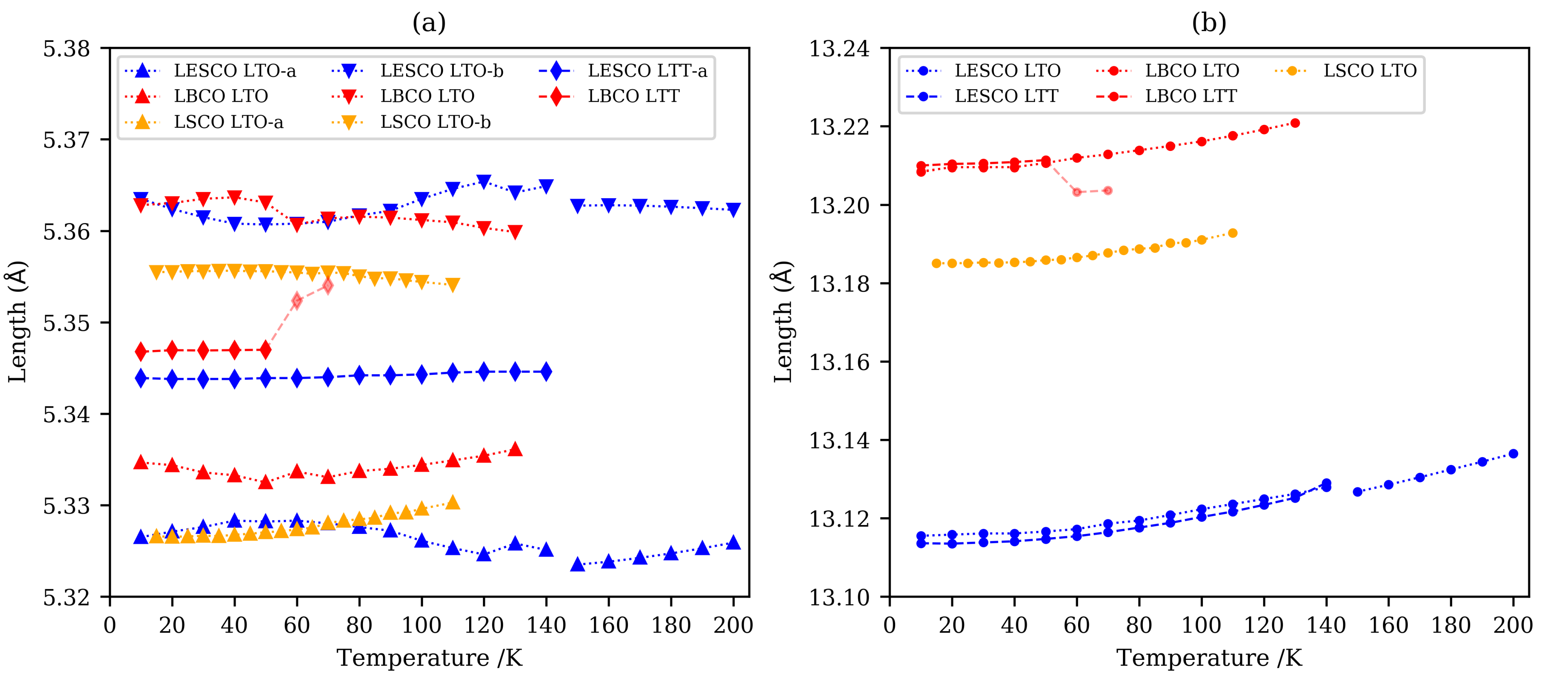


**Figure 12**. Plots of the (a) *a* and *b*, and (b) *c* cell parameters for the LTO and LTT phases of LESCO, LBCO and LSCO powder diffraction data as a function of temperature.


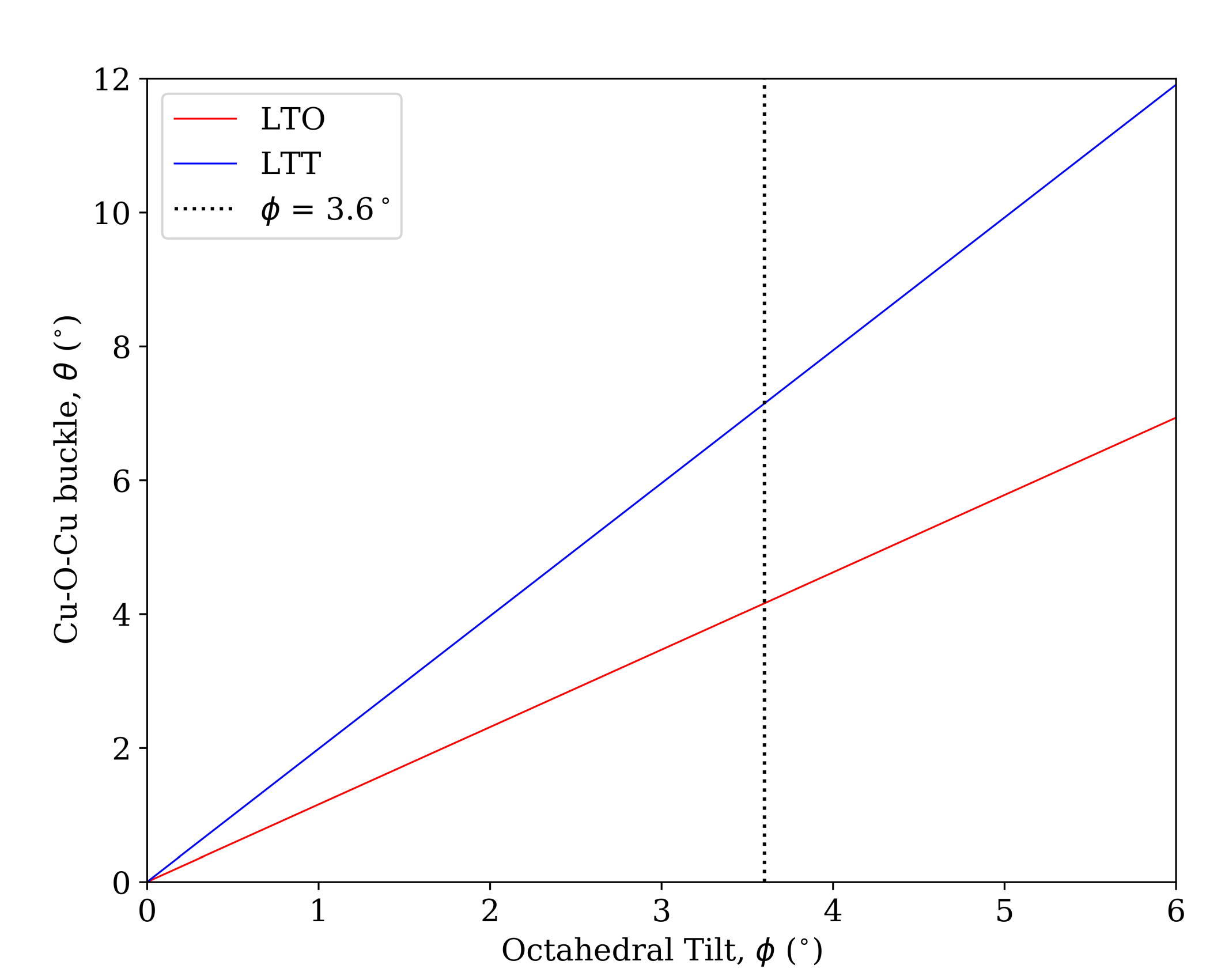


**Figure 13.** Plot of the different Cu-O-Cu buckle angle, *θ*, in the LTT and LTO phases as a function of octahedral tilt, *φ*, extrapolated from the experimental models. We place a dotted black line to highlight the critical tilt value, *φ*_c_ = 3.6 °, as discussed in the literature.

**Table** **2** Table presenting the atomic coordinates with calculated standard uncertainties for where either the LTT or LTO components of the LTO+LTT model are instead refined as an LTLO phase for LESCO. Atoms and the relevant coordinates which break (beyond 3σ confidence) their high-symmetry constraint are highlighted in red while those which comply within 3σ are in green; coordinates and atoms of no consequence are grey.

| **T (K)** | **Atomic site** | **x (frac)** | **y (frac)** | **z (frac)** |
| --- | --- | --- | --- | --- |
| 30 | A-site (LTO) | 0.0006(6) | 0.0065(4) | 0.3603(2) |
|  | O1 (LTO) | -0.0061(17) | -0.0270(5) | 0.1833(3) |
|  | O2 (LTO) | 0.25 | 0.25 | 0.0093(8) |
|  | O3 (LTO) | -0.25 | 0.25 | 0.0008(6) |
|  | A-site (LTT) | 0.0067(4) | 0.0069(5) | 0.3602(2) |
|  | O1 (LTT) | -0.0313(16) | -0.0331(17) | 0.1828(3) |
|  | O2 (LTT) | 0.25 | 0.25 | 0.0149(5) |
|  | O3 (LTT) | -0.25 | 0.25 | -0.0037(6) |
|  |  |  |  |  |
| 70 | A-site (LTO) | -0.0015(6) | 0.0060(4) | 0.3617(2) |
|  | O1 (LTO) | 0.0005(13) | -0.0264(4) | 0.1835(3) |
|  | O2 (LTO) | 0.25 | 0.25 | 0.0066(8) |
|  | O3 (LTO) | -0.25 | 0.25 | 0.0035(8) |
|  | A-site (LTT) | 0.0065(5) | 0.0076(6) | 0.3619(3) |
|  | O1 (LTT) | -0.0345(14) | -0.0389(13) | 0.1828(3) |
|  | O2 (LTT) | 0.25 | 0.25 | 0.0167(5) |
|  | O3 (LTT) | -0.25 | 0.25 | -0.0021(11) |
|  |  |  |  |  |
| 100 | A-site (LTO) | -0.0027(6) | 0.0062(4) | 0.3598(2) |
|  | O1 (LTO) | 0.0017(12) | -0.0268(4) | 0.1837(3) |
|  | O2 (LTO) | 0.25 | 0.25 | 0.0053(6) |
|  | O3 (LTO) | -0.25 | 0.25 | 0.0043(7) |
|  | A-site (LTT) | 0.0052(4) | 0.0141(6) | 0.3623(3) |
|  | O1 (LTT) | -0.0362(13) | -0.0383(13) | 0.1826(3) |
|  | O2 (LTT) | 0.25 | 0.25 | 0.0174(5) |
|  | O3 (LTT) | -0.25 | 0.25 | -0.0008(11) |
|  |  |  |  |  |
| 130 | A-site (LTO) | 0.0048(4) | 0.0111(4) | 0.35976(19) |
|  | O1 (LTO) | 0.0008(11) | -0.0268(4) | 0.1834(3) |
|  | O2 (LTO) | 0.25 | 0.25 | 0.0035(6) |
|  | O3 (LTO) | -0.25 | 0.25 | 0.0077(5) |
|  | A-site (LTT) | 0.0075(4) | 0.0093(7) | 0.3619(4) |
|  | O1 (LTT) | -0.0346(17) | -0.0407(14) | 0.1827(3) |
|  | O2 (LTT) | 0.25 | 0.25 | 0.0175(8) |
|  | O3 (LTT) | -0.25 | 0.25 | 0.0065(15) |


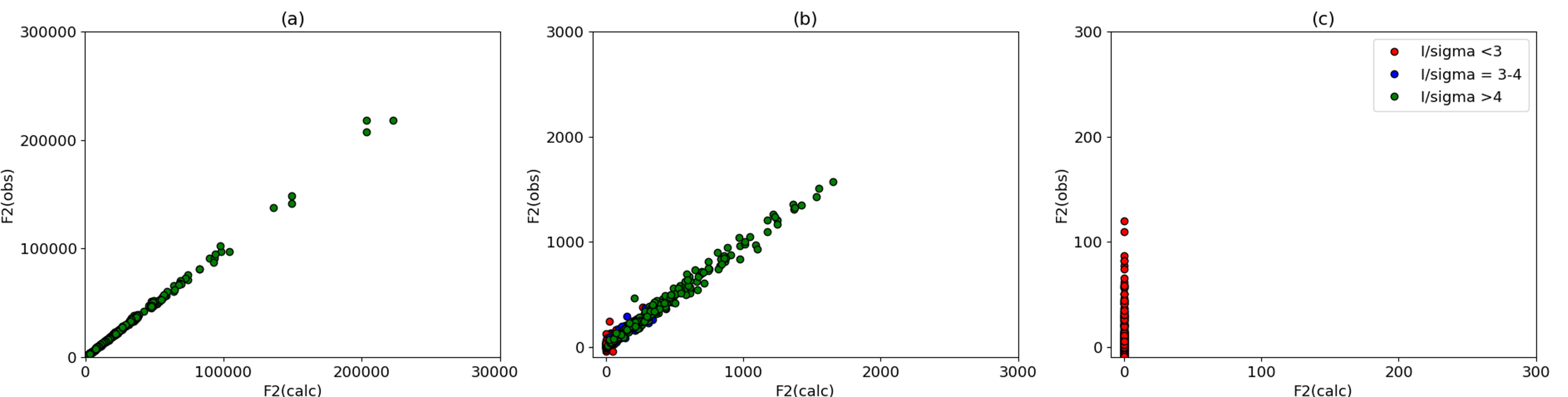


**Figure 14.** Plots of the observed and calculated structure factors corresponding to (a) F_HTT_, (b) SS_common_ and (c) SS_LTT_ for an LTO-only refinement against the single-crystal data of LSCO at 30 K. Note that for SS_LTT_ (*i.e.*, those reflections corresponding to violations of twinned-*Bmab* systematic absences otherwise seen in the *P*-centred phases, LTT and LTLO), the observed structure factors constitute only noise, *i.e.*, I_obs_<3σ(I_obs_) (compare with Supplemental Information Figures 5-10).


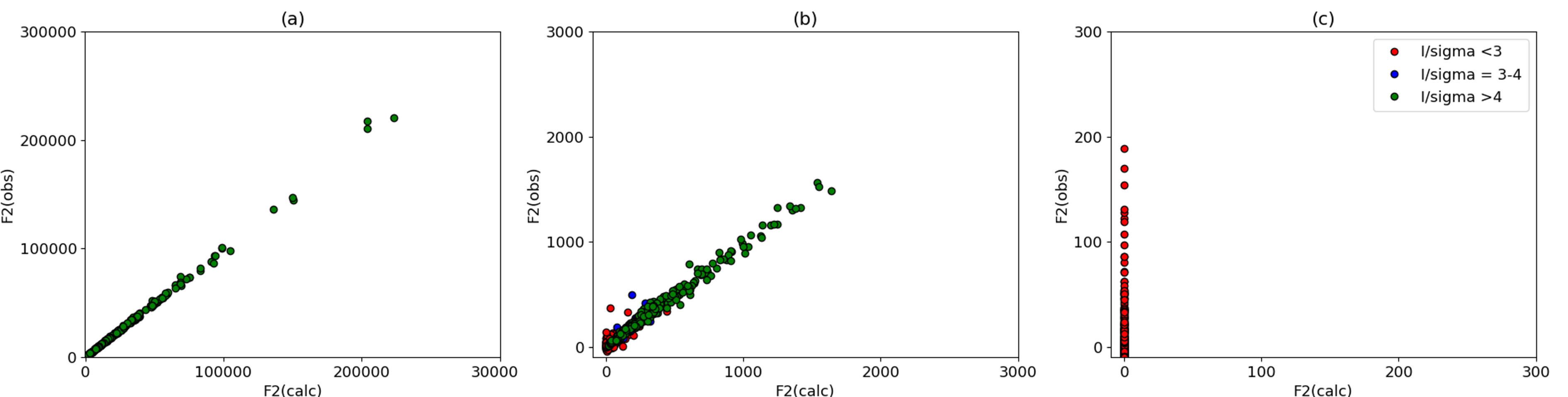


**Figure 15.** Plots of the observed and calculated structure factors corresponding to (a) F_HTT_, (b) SS_common_ and (c) SS_LTT_ for an LTO-only refinement against the single-crystal data of LSCO at 50 K. Note that for SS_LTT_ (*i.e.*, those reflections corresponding to violations of twinned-*Bmab* systematic absences otherwise seen in the *P*-centred phases, LTT and LTLO), the observed structure factors constitute only noise, *i.e.*, I_obs_<3σ(I_obs_) (compare with Supplemental Information Figures 5-10).

**References**

1. Nowell, H., Barnett, S. A., Christensen, K. E., Teat, S. J. & Allan, D. R. I19, the small-molecule single-crystal diffraction beamline at Diamond Light Source. *J. Synchrotron Radiat.* **19**, 435–441 (2012).

2. Campbell, B. J., Stokes, H. T., Tanner, D. E. & Hatch, D. M. ISODISPLACE: A web-based tool for exploring structural distortions. *J. Appl. Crystallogr.* **39**, 607–614 (2006).

3. Stokes, H. T., Hatch, D. M. & Campbell, B. J. ISODISTORT, ISOTROPY Software Suite. iso.byu.edu.
